# Maternal opioid prescriptions and subsequent risk of hospital admission in offspring in South Korea: A nationwide birth cohort study

**DOI:** 10.1371/journal.pmed.1004829

**Published:** 2026-10-13

**Authors:** Hyeon Jin Kim, Yesol Yim, Christa J. Nehs, Jaeyu Park, Eun Soo Kim, Sung Joon Cho, Guillaume Fond, Laurent Boyer, Jiseung Kang, Dong Keon Yon

**Affiliations:** 1 Center for Digital Health, Medical Science Research Institute, Kyung Hee University College of Medicine, Seoul, South Korea; 2 Department of Precision Medicine, Kyung Hee University College of Medicine, Seoul, South Korea; 3 Division of Sleep Medicine, Harvard Medical School, Boston, Massachusetts, United States of America; 4 Mass General Brigham Department of Anesthesiology, Massachusetts General Hospital, Boston, Massachusetts, United States of America; 5 Department of Psychiatry, Kangbuk Samsung Hospital, Sungkyunkwan University School of Medicine, Seoul, South Korea; 6 CEReSS-Health Service Research and Quality of Life Center, Aix Marseille University, Marseille, France; 7 CEReSS-Health Service Research and Quality of Life Center, Assistance Publique-Hopitaux de Marseille, Aix Marseille University, Marseille, France; 8 School of Health and Environmental Science, College of Health Science, Korea University, Seoul, South Korea; 9 Department of Health and Safety Convergence Science, Graduate School, Korea University, Seoul, South Korea; 10 Department of Pediatrics, Kyung Hee University Medical Center, Kyung Hee University College of Medicine, Seoul, South Korea; University of Cambridge School of Clinical Medicine, UNITED KINGDOM OF GREAT BRITAIN AND NORTHERN IRELAND

## Abstract

**Background:**

Prenatal opioid exposure may disrupt infant development and immune system function, potentially increasing long-term susceptibility to hospital admissions. Thus, we aimed to investigate the association between prenatal opioid exposure and the risk of hospital admission in children.

**Methods and findings:**

We established a population-based birth cohort in South Korea by linking mothers and children using National Health Insurance Service data and followed from January 1, 2009, to December 31, 2023. We constructed propensity score (PS)-matched and sibling comparison cohorts and performed a multi-pronged approach, including dose-dependent and time-attenuation analyses. All 5,249,429 individuals with eligible children born between January 1, 2010, and December 31, 2017, were included (2,236,167 mothers and 3,013,262 children). Infants were followed up for up to 14 years, depending on birth year. Prenatal exposure to opioids was defined as maternal receipt of two or more prescriptions within any given trimester. Adjusted incidence rate ratio (aIRR) of hospital admission among children with prenatal opioid exposure was estimated for causes including infections, non-infectious respiratory conditions, non-infectious gastrointestinal conditions, oral cavity diseases, injuries/external causes, and non-infectious genitourinary conditions. Analyses were adjusted for maternal age at delivery, infant sex, region of residence, household income level, birth season, parity, severe maternal morbidity score, number of outpatient visits and hospital admissions in the year before pregnancy, preterm birth, low birth weight, delivery type, year of delivery, maternal history of substance use and neuropsychiatric disorders, and plurality. Of the 2,928,186 children, we identified 2,809,515 (51.4% male) and 118,671 (51.3% male) infants without and with prenatal opioid exposure, respectively. The rate of hospital admissions in children with prenatal opioid exposure was 28% higher in the PS-matched cohort (aIRR 1.28 [95% CI 1.28, 1.29]; *p* < 0.001). The association was attenuated in the sibling comparison cohort, indicating the potential influence of shared familial factors, but remained statistically significant (aIRR 1.08 [95% CI 1.07, 1.10]; *p* < 0.001). Hospital admission rates in children increased in a dose-dependent manner with maternal opioid dose and duration of opioid prescriptions (low-dose exposure: aIRR 1.19 [95% CI 1.19, 1.20]; *p* < 0.001; high-dose exposure: aIRR 1.40 [95% CI 1.39, 1.41]; *p* < 0.001). Prenatal opioid exposure was significantly associated with increased rates of hospital admission across several causes of morbidities, including infections, respiratory non-infections, oral cavity, gastrointestinal non-infections, genitourinary non-infections, and injury/external conditions. These associations between prenatal opioid exposure and hospital admission rates in children diminished with age but remained evident up to five years of age. The main limitations of this study’s methodology include the use of prescription records, which may not fully capture actual medication intake, and residual confounding that cannot be entirely excluded.

**Conclusions:**

In this nationwide birth cohort study, maternal opioid prescription during pregnancy was associated with a substantial increase in the risk of hospital admissions in the offspring across various causes of morbidities. Although the attenuated estimates in the sibling comparison analysis suggest that familial factors may partly explain this association, these findings may inform postnatal monitoring and management strategies for children born to mothers who received opioid prescriptions during pregnancy.

## Author summary

**Why was this study done?:**

- Prenatal opioid exposure may disrupt infant development and immune system function, potentially increasing long-term susceptibility to hospital admissions.
- Prior studies often had limitations such as unmeasured confounding factors, limited sample sizes, or restricted follow-up durations, leaving uncertainty about the long-term impacts of prenatal opioid exposure on child health.
- This study aimed to investigate the association between maternal opioid prescriptions during pregnancy and the subsequent risk of hospital admissions in children using a nationwide birth cohort in South Korea.

**What did the researchers do and find?:**

- Using nationwide data from the South Korean National Health Insurance Service, the researchers followed 2,928,186 children born between 2010 and 2017 for up to 14 years.
- Children with prenatal opioid exposure, defined as maternal receipt of two or more opioid prescriptions during pregnancy, had a 28% higher rate of hospital admissions than unexposed children, showing a dose-dependent association.
- These associations persisted in sibling comparison analyses and were most evident up to 5 years of age across several causes of morbidity.

**What do these findings mean?:**

- Maternal opioid prescriptions during pregnancy were associated with an increased risk of hospital admissions in children, showing a dose-dependent and partially persistent pattern up to five years of age.
- These findings suggest the need for additional postnatal health checks for those born to mothers with opioid prescriptions during pregnancy.
- However, these findings should be interpreted with caution, as the study relied on electronic health records, which may not reflect actual use of medication, and unmeasured factors shared within families may still partly explain the observed associations.

## Introduction

Over the past three decades, the global opioid crisis has generated an extensive burden, resulting in substantial morbidity and mortality [1]. Opioid prescription and prescription dose have continued to rise in clinical practice, and a considerable proportion of pregnant women are exposed to these drugs [1]. In the United States, using administrative data showed that 14%–22% of women filled a prescription for opioids during pregnancy [2]. Furthermore, the recent Pregnancy Risk Assessment Monitoring System survey from the US Centers for Disease Control and Prevention (CDC) reported that about 6.6% of women self-reported using prescription opioids during pregnancy, with approximately one in five of these women indicating non-medical use, such as obtaining opioids from non-healthcare sources or using for reasons other than pain [2]. In South Korea, approximately 6.9% of infants born from 2009 to 2020 were exposed to prenatal opioids during gestation following maternal opioid prescriptions [3].

Pregnant women are frequently cautious about medication use during pregnancy, yet certain clinical conditions necessitate opioid analgesics [4,5]. Unique physiological changes in pregnancy, including increased ligamentous laxity, altered weight bearing, and heightened pain sensitivity, can precipitate or aggravate various pains such as lower back pain, sometimes requiring management with opioid pain relievers [4–6]. Indeed, opioids are prescribed during pregnancy not only for chronic or severe pain but also occasionally for acute conditions, such as codeine-based cough suppressants for persistent coughing [7]. Such uses reflect the delicate balance between managing maternal symptoms and minimizing fetal exposure. Given this balance, clinicians may face difficult decisions about opioid therapy in pregnancy, especially as the prevalence of indicated pain conditions rises during gestation [8].

There is growing concern about the potential indirect impacts of prenatal opioid exposure on health outcomes in children [9]. Exposure to opioids in utero, especially in the first trimester and/or with high doses, has been associated with adverse developmental and behavioral consequences in children [3,10]. Previous systematic reviews from the US CDC raised alarms about possible teratogenic effects of opioid use in pregnancy, reporting an increased risk of congenital anomalies, including congenital heart defects, neural tube defects, orofacial clefts, and clubfoot, in opioid-exposed infants [10].

However, it is also recognized that maternal opioid treatments can be clinically necessary, especially in cases of refractory chronic pain or severe obstetric complications, and thus their potential therapeutic benefits cannot be entirely discounted [10]. Importantly, recent large-scale birth cohort studies have not found strong evidence of teratogenicity or subsequent neuropsychiatric harm to offspring from moderate maternal opioid use during pregnancy. When high-risk conditions such as first-trimester exposure or high opioid prescription doses are avoided, prescription opioid use in pregnancy has not been associated with an increase in the risk of major congenital malformations or in the risk of neuropsychiatric disorders in the offspring [3,10]. Therefore, the association between opioid exposure during gestation and child health outcomes remains a critical area of investigation.

Prior studies often had limitations such as unmeasured confounding factors or limited sample sizes, leaving residual uncertainty about more subtle or long-term associations with outcomes in children [11,12]. A more definitive assessment using a nationwide, population-based birth cohort could provide needed clarity. Outcome categories, including infection, respiratory, gastrointestinal, genitourinary, oral cavity, and injury/external causes, were defined in accordance with a prior nationwide birth cohort study utilizing the same database, which established clinically meaningful classifications for childhood hospital admissions in South Korea [13]. Given these gaps in the literature, our study may help inform recommendations for routine postnatal health surveillance of children born to mothers who required opioids during pregnancy. Specifically, we aimed to examine the association between maternal opioid prescriptions during pregnancy and the subsequent risk of hospital admissions in children, utilizing a large-scale birth cohort in South Korea.

## Methods

### General approach

This study did not have a formal prospective protocol or pre-registered analysis plan. The analytic approach was determined prior to data analysis based on the study objectives and methods established in previous research using the same database. No data-driven changes to the planned analyses were made [3,13]. To explore the potential association between prenatal opioid exposure and the incidence rate of hospital admissions in children, we employed a multi-pronged analytic strategy that involved three distinct approaches to comprehensively examine aspects of hospital admissions. First, we used the incidence rate ratio (IRR) to compare the frequency of hospital admissions over the entire follow-up period [14,15]. This approach assesses both the initial risk and the persistence of healthcare use over time, which were clinically relevant for understanding the burden of illness and care patterns [14,15]. Second, to evaluate whether the association followed a dose-dependent manner, we calculated the cumulative opioid dose during pregnancy and assessed the relationship between the level of opioid dose and hospital admission [3]. Third, to examine the potential time-attenuated association, we estimated IRRs across different child age intervals [13]. This aimed to investigate the persistence of the association over time.

### Data source

In this large-scale, nationwide, population-based cohort study, we used data from the National Health Insurance Service (NHIS) of South Korea [16]. The NHIS was a government-affiliated agency under the Ministry of Health and Welfare that operated a national, claim-based health insurance system covering approximately 98% of the Korean population [16]. The NHIS database included detailed information on all insured individuals in South Korea, including sociodemographic characteristics, clinical diagnosis records based on the International Classification of Diseases (ICD) codes, medical procedures, prescription records, and health examinations.

### Ethics statement

This study was approved by the Institutional Review Board of Kyung Hee University (KHSIRB-24-511[EA]), which waived the requirement for individual informed consent because the study used de-identified administrative data. Access to the data was obtained from the Korean NHIS following review and approval of the data use application (NHIS-2025-03-1-040). All patient-related information, including personal identification numbers, was anonymized by the Korean government prior to release. The study followed the Reporting of studies Conducted using Observational Routinely-collected health Data (RECORD) statement, with the completed checklist provided as Supporting Information [17].

### Study design and participants

This study used a birth cohort to investigate the association between maternal drug use during pregnancy and health outcomes in children. The cohort was restricted to pregnancies resulting in live births, as only live-born children could contribute follow-up time for health outcomes in children. The study population comprised 5,249,429 individuals, including 3,013,262 children born between January 1, 2010, and December 31, 2017, and their 2,236,167 mothers. Mother and child pairs were linked using unique family identification numbers, enabling siblings and multiple births (e.g., twins) to be distinguished while treating each child as a separate individual [18]. Medical records of participants were followed from January 1, 2009, including a one-year look-back period prior to pregnancy for mothers, through December 31, 2023.

Participants were excluded based on the following criteria: (1) missing information on socioeconomic status; (2) missing birth date; (3) children diagnosed with immunodeficiency disorders, cystic fibrosis, chronic kidney disease, β-thalassemia, sickle-cell disorders, or cancer; (4) children who were hospitalized within one month of birth; and (5) mothers whose offspring were already excluded. Since complex chronic conditions and health status requiring hospitalization shortly after birth are independently associated with frequent hospital admission, criteria (3) and (4) were applied to reduce potential confounding in outcome assessment [19]. No cases of neonatal opioid withdrawal syndrome were identified in this cohort, consistent with the prescription-based nature of opioid use in South Korea [20,21]. After excluding 85,076 children and 48,589 mothers, the final cohort consisted of 2,928,186 children and 2,187,578 mothers (Fig 1).

**Fig 1:**
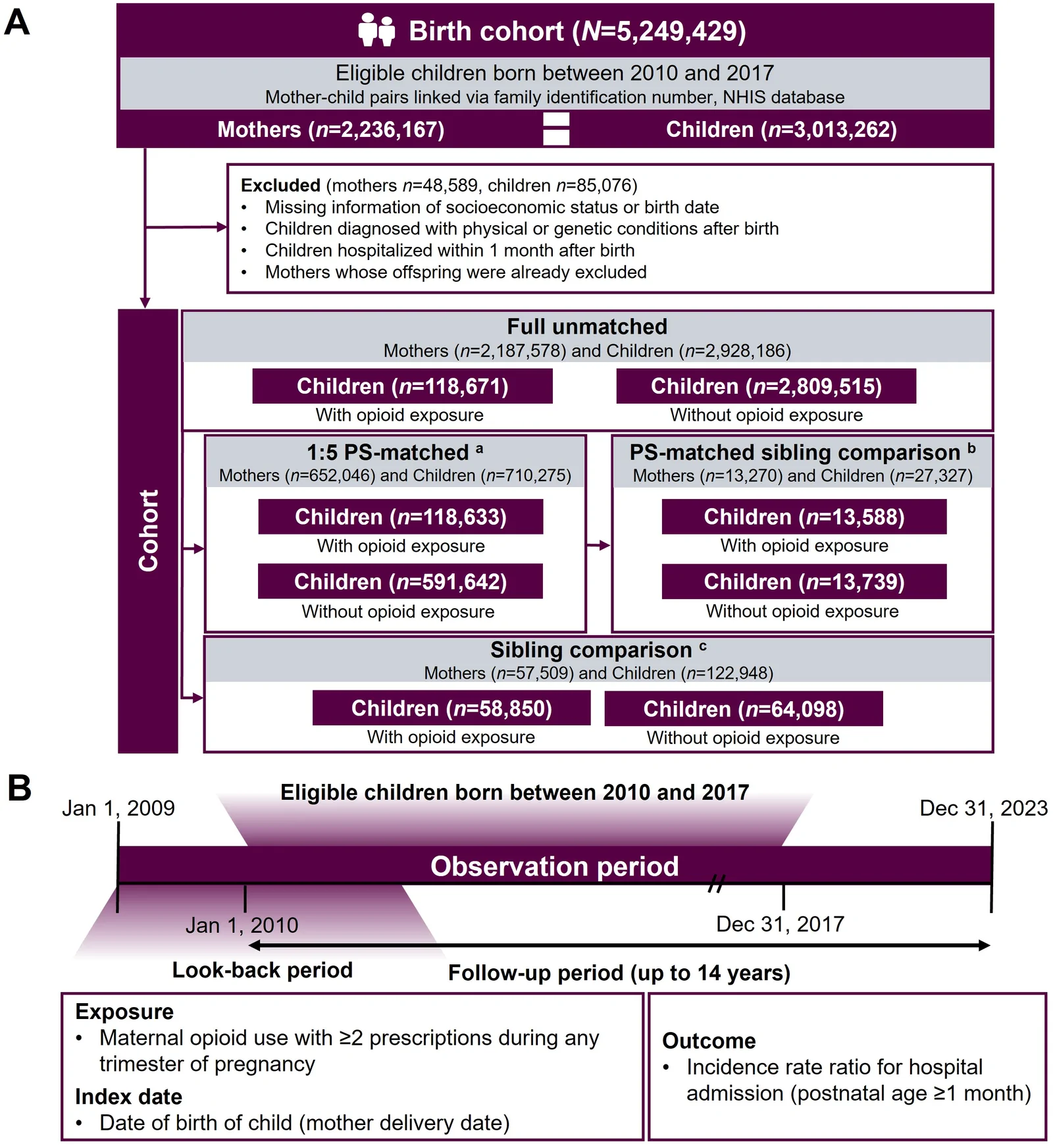
Study design mother-child paired cohort in South Korea. *Abbreviation:* PS, propensity score. ^a^1:5 PS-matched cohort was constructed from the full unmatched cohort, pairing exposed and unexposed groups in a 1:5 ratio using PS. ^b^PS-matched sibling comparison cohort, derived from the 1:5 PS-matched cohort. ^c^Sibling comparison cohort, identified from the full unmatched cohort.

### Exposure

In accordance with previous research, prenatal opioid exposure was defined as maternal receipt of two or more opioid prescriptions within any given trimester; mothers with fewer than two prescriptions were classified as unexposed [3,9]. Opioid exposure was identified using NHIS prescription claims for codeine, dihydrocodeine, fentanyl, morphine, oxycodone, and tramadol. Based on this definition, the exposure group was subsequently classified into subgroups according to the following factors: (1) trimester of exposure (first, second, third, and more than one trimester); (2) cumulative opioid dose during pregnancy calculated in morphine milligram equivalents (MME), with median-based classification (low-dose, ≤18 MME; high-dose, >18 MME); (3) total number of prescription days (1–7, 8–11, and ≥12 days); (4) number of opioid prescriptions (2 and ≥3); (5) maternal health condition associated with opioid prescriptions (pain-related [including neoplasms, diseases of the blood and blood-forming organs, certain disorders involving the immune mechanism, endocrine, nutritional and metabolic diseases, neuropsychiatric disorders, diseases of the nervous system, diseases of the ear and mastoid process, diseases of the circulatory system, diseases of digestive system, diseases of the skin and subcutaneous tissue, diseases of musculoskeletal system and connective tissue, and diseases of the genitourinary system], antitussive-related [including infectious and parasitic diseases and diseases of the respiratory system], and others); and (6) specific opioid (codeine, dihydrocodeine, fentanyl, morphine, oxycodone, tramadol, and polytherapy) [3]. Polytherapy was defined as concurrent use of two or more distinct opioids during the exposure period.

### Outcome

The study outcome was the number of hospital admission cases among children with prenatal opioid exposure, resulting from diagnoses under specific clinical diagnosis (infection, respiratory non-infection, gastrointestinal non-infection, oral cavity, injury/external, or genitourinary non-infection) [13]. The “non-infection” categories include diagnoses within the respective organ systems that exclude infectious causes. Hospital admissions were identified when diagnosis and admission claim occurred concurrently during follow-up beginning one month after birth. Diagnoses were defined using ICD-10 codes, and admissions were based on claim codes specified in the NHIS database (Table A in S1 Appendix).

### Covariates

Mother covariates included maternal age at delivery, region of residence (rural and urban) [22], income level (1st to 4th quartile categories), severe maternal morbidity (SMM) score identified using the ICD code-based definition introduced by Bateman and colleagues (Table A in S1 Appendix) [23,24], hospital admission and outpatient visit during the year prior to pregnancy, and history of substance use disorders and neuropsychiatric disorders [25,26]. Child characteristics considered in the study were sex, preterm birth (≤36 weeks), and low birth weight (≤2,499g). Delivery-related factors included parity (nulliparous and multiparous), delivery type (vaginal delivery and cesarean section), birth season (spring [March to May], summer [June to August], autumn [September to November], and winter [December to February]), year of delivery (2010–2012, 2013–2015, and 2016–2017), and plurality (singleton and multiple birth). All covariates were derived from eligibility data and claim codes provided by the NHIS.

### Propensity score-matched cohort

To control for potential confounders between the two groups according to prenatal opioid exposure status, a propensity score (PS)–matched cohort was constructed from the full cohort [27]. PS was estimated using a logistic regression model that included maternal age at delivery, region of residence, income level, parity, SMM score, delivery type, number of hospital admissions and outpatient visits in the year before pregnancy, maternal history of substance use disorders and neuropsychiatric disorders, infant sex, birth season, year of delivery, and at-risk newborn (preterm birth and low birth weight) as covariates. Matching was performed at a 1:5 ratio using a “greedy nearest neighbor” algorithm with a caliper of 0.001, individuals from the two groups having PS differences less than the caliper being randomly matched [28]. The adequacy of matching was assessed using the standardized mean difference (SMD), with values below 0.1 indicating no significant imbalance between two groups (Fig A in S1 Appendix). As a result, 118,633 opioid-exposed children were matched with 591,642 unexposed children.

### Sibling comparison cohort

To mitigate potential biases from unmeasured confounders, including those related to genetics, lifestyle, and environmental influences, a sibling comparison cohort derived from the full cohort was established as a robust analytic strategy [3]. In this cohort, only sibships containing at least one exposed and one unexposed child were included, enabling within-sibling comparisons under the control of shared familial characteristics. Accordingly, children without siblings (excluded families *n* = 1,505,851) or from families in which all siblings shared the same exposure status (excluded families *n* = 624,218) were excluded. In addition, a family-level stratification variable was constructed to account for within-family comparisons. A total of 58,850 children with opioid exposure and 64,098 children without opioid exposure from 57,509 families were included in this sibling comparison cohort.

### Statistical analysis

To examine the association between prenatal opioid exposure and hospital admissions in children, IRRs were estimated using hospital admission rates per person-year during the follow-up period. Each child was observed from birth until death or the end of the observation period. IRR with 95% confidence intervals (CIs) were estimated using a generalized linear model with a Poisson distribution, incorporating person-year as an offset through a log link function [14,15]. To account for potential confounding, we used two adjusted models. Model 1 was adjusted for maternal age at delivery, infant sex, region of residence, household income level, birth season, parity, SMM score, number of outpatient visits and hospital admissions in the year before pregnancy, preterm birth, low birth weight, delivery type, and year of delivery. Model 2 was adjusted for all variables in Model 1, with further adjustment for maternal history of substance use disorders and neuropsychiatric disorders and plurality. In addition to the adjusted models, unadjusted (crude) IRRs estimated from models containing prenatal opioid exposure only are presented in all tables. To facilitate interpretation of the magnitude and clinical relevance of the findings, we calculated incident rates per 1,000 person-years in the exposed and unexposed groups and the corresponding rate differences. These measures quantify the absolute increase in the rate of hospital admissions associated with prenatal opioid exposure. In addition, relative aIRR was estimated across diagnostic categories of hospital admissions to assess differences in the associations between prenatal opioid exposure and hospital admissions.

Additional subgroup and sensitivity analyses along with a PS-matched cohort and sibling comparison cohort were conducted. First, for each outcome, we examined dose-dependent associations by analyzing opioid dosage levels during pregnancy to explore how variation in exposure intensity corresponded with hospital admission rates [3]. Second, stratification analysis with child age groups (0–1, 2–3, 4–5, 6–7, 8–10, and 11–14 years) across each outcome was conducted to evaluate the associations at different time points. Third, a PS-matched sibling comparison cohort was constructed to further account for shared familial factors beyond those accounted for by PS matching. Fourth, the adjusted models were re-estimated after excluding preterm birth, low birth weight, and delivery type from the adjustment variables, as these factors may serve as potential mediators between prenatal opioid exposure and child outcomes. Fifth, under the primary definition, prenatal opioid exposure was defined as maternal receipt of 2 or more opioid prescriptions within any given trimester [3], so pregnancies with only a single opioid prescription were classified as unexposed. In a sensitivity analysis, we therefore excluded these pregnancies so that the reference group comprised only pregnancies with no opioid prescription. PS matching was repeated, and discordant sibships were reidentified after the exclusion. All statistical tests were evaluated using a two-sided significance level of *p* < 0.05. All analyses were conducted using SAS software (version 9.4; SAS Institute, Cary, NC, USA).

### Artificial intelligence statement

We confirm that no artificial intelligence tools or technologies were used in the conduct of the study or in the preparation of the article content.

## Results

Of the 2,928,186 infants, we identified 95.9% (2,809,515/2,928,186) infants without prenatal opioid exposure (51.4% male) and 4.1% (118,671/2,928,186) infants with prenatal opioid exposure (51.3% male) in the full unmatched cohort within follow-up periods from January 1, 2010, to December 31, 2023 (Fig 1). Demographic and baseline characteristics are detailed in Table 1 and Table B in S1 Appendix. After 1:5 PS matching, the SMD values were less than 0.1, indicating no major imbalances in the baseline characteristics (Table 1 and Fig A in S1 Appendix).

**Table 1:** Baseline characteristics of study participants from 2010 to 2017.

	Full unmatched cohort^a^ (*n* = 2,928,186)	1:5 PS-matched cohort^b^		
		(*n* = 710,275)		
		Children without prenatal exposure	Child with prenatal opioid exposure	SMD^*^
		(*n* = 591,642)	(*n* = 118,633)	
**Maternal characteristics**				
Maternal age at delivery year, mean (SD)	31.9 (4.2)	32.4 (4.1)	32.3 (4.2)	<0.01
Maternal age at delivery year, *n* (%)				<0.01
≤19	7,516 (0.3)	1,369 (0.2)	342 (0.3)	
20–24	128,226 (4.4)	20,778 (3.5)	4,468 (3.8)	
25–29	636,816 (21.7)	110,470 (18.7)	22,212 (18.7)	
30–34	1,416,274 (48.4)	283,477 (47.9)	56,579 (47.7)	
≥35	739,354 (25.2)	175,548 (29.7)	35,032 (29.5)	
Region of residence, *n* (%)				<0.01
Urban	1,298,945 (44.4)	257,155 (43.5)	51,644 (43.5)	
Rural	1,629,241 (55.6)	334,487 (56.5)	66,989 (56.5)	
Income level, *n* (%)				0.03
1st quartile	719,854 (24.6)	153,388 (25.9)	30,749 (25.9)	
2nd quartile	696,769 (23.8)	139,900 (23.6)	28,040 (23.6)	
3rd quartile	869,869 (29.7)	174,502 (29.5)	35,001 (29.5)	
4th quartile	641,694 (21.9)	123,852 (20.9)	24,843 (20.9)	
Parity, *n* (%)				<0.01
1	1,911,449 (65.3)	261,909 (44.3)	53,144 (44.8)	
≥2	1,016,737 (34.7)	329,733 (55.7)	65,489 (55.2)	
SMM score, *n* (%)				0.03
0	2,705,904 (92.4)	537,185 (90.8)	106,752 (90.0)	
1	214,293 (7.3)	52,454 (8.9)	11,333 (9.6)	
≥2	7,989 (0.3)	2,003 (0.3)	548 (0.5)	
Delivery type, *n* (%)				0.01
Vaginal delivery	1,711,877 (58.5)	318,095 (53.8)	63,196 (53.3)	
Cesarean section	1,216,309 (41.5)	273,547 (46.2)	55,437 (46.7)	
Number of hospital admissions in a year before pregnancy, *n* (%)				0.05
0	2,452,868 (83.8)	466,194 (78.8)	92,671 (78.1)	
1	381,463 (13.0)	98,984 (16.7)	20,011 (16.9)	
≥2	93,855 (3.2)	26,464 (4.5)	5,951 (5.0)	
Number of outpatient contacts in a year before pregnancy, *n* (%)				<0.01
0	192,625 (6.6)	13,150 (2.2)	2,694 (2.3)	
1	158,012 (5.4)	12,684 (2.1)	2,629 (2.2)	
≥2	2,577,549 (88.0)	565,808 (95.6)	113,310 (95.5)	
History of neuropsychiatric disorder, *n* (%)				0.02
No	2,700,853 (92.2)	519,552 (87.8)	103,344 (87.1)	
Yes	227,333 (7.8)	72,090 (12.2)	15,289 (12.9)	
History of substance use disorder, *n* (%)				0.01
No	2,924,140 (99.9)	590,475 (99.8)	118,326 (99.7)	
Yes	4,046 (0.1)	1,167 (0.2)	307 (0.3)	
**Infant characteristics**				
Infant sex, *n* (%)				0.01
Male	1,503,541 (51.3)	302,880 (51.2)	60,839 (51.3)	
Female	1,424,645 (48.7)	288,762 (48.8)	57,794 (48.7)	
Birth season, *n* (%)				<0.01
Spring	756,601 (25.8)	137,462 (23.2)	27,616 (23.3)	
Summer	717,720 (24.5)	161,937 (27.4)	32,403 (27.3)	
Autumn	723,695 (24.7)	146,591 (24.8)	29,454 (24.8)	
Winter	730,170 (24.9)	145,652 (24.6)	29,160 (24.6)	
Year of delivery, *n* (%)				0.02
2010–2012	1,202,856 (41.1)	196,400 (33.2)	39,404 (33.2)	
2013–2015	1,095,753 (37.4)	209,394 (35.4)	42,192 (35.6)	
2016–2017	629,577 (21.5)	185,848 (31.4)	37,037 (31.2)	
At-risk newborn, *n* (%)				
Preterm birth	107,211 (3.7)	25,068 (4.2)	6,142 (5.2)	0.04
Low birth weight	85,857 (2.9)	17,794 (3.0)	4,493 (3.8)	0.04

*SMD <0.1 corresponds to no major imbalance.

^a^Full unmatched cohort involves infants born between 2010 and 2017.

^b^1:5 PS-matched cohort, derived from the full unmatched cohort, pairs the exposed and unexposed groups in a 1:5 ratio using PS.

*Abbreviation:* PS, propensity score; SD, standard deviation; SMD, standardized mean difference; SMM, severe maternal morbidity.

The hospital admission rate was 28% higher in children with prenatal opioid exposure compared to those without prenatal opioid exposure in the PS-matched cohort (adjusted IRR [aIRR] 1.28 [95% CI 1.28, 1.29]; *p* < 0.001; Table 2). In particular, exposure to opioids during more than one trimester showed significantly higher rates of hospital admission in the offspring compared with non-exposed offspring (aIRR 1.48 [95% CI 1.46, 1.50]; *p* < 0.001). Rates of hospital admission in children with prenatal opioid exposure increased in a dose-dependent manner (low-dose exposure: aIRR 1.19 [95% CI 1.19, 1.20]; *p* < 0.001; high-dose exposure: aIRR 1.40 [95% CI 1.39, 1.41]; *p* < 0.001). Compared with non-exposed infants, hospital admission rates showed increasing trends depending on the days of opioid prescriptions (1–7 days of opioid prescriptions: aIRR 1.22 [95% CI 1.21, 1.22; *p* < 0.001]; 8–11 days: aIRR 1.30 [95% CI 1.29, 1.31; *p* < 0.001]; ≥ 12 days: aIRR 1.42 [95% CI 1.41, 1.43; *p* < 0.001]) and number of opioid prescriptions (2 times of opioid prescriptions: aIRR 1.22 [95% CI 1.21, 1.22; *p* < 0.001]; ≥ 3 times: aIRR 1.36 [95% CI 1.35, 1.37; *p* < 0.001]). Pain-related reasons for maternal opioid prescriptions showed slightly higher rates of hospital admission in their children compared to antitussive-related prescriptions of opioids during pregnancy, although both had increased rates compared to non-exposed children (pain-related: aIRR 1.33 [95% CI 1.32, 1.35; *p* < 0.001]; antitussive-related: aIRR 1.26 [95% CI 1.25, 1.27; *p* < 0.001]). When stratified by opioid type, hospital admission rates in offspring also varied according to the specific opioid prescribed during pregnancy. Compared with non-exposed infants, exposure to morphine was associated with the highest rate of hospital admissions (aIRR 1.62 [95% CI 1.57, 1.68]; *p* < 0.001), followed by opioid polytherapy (aIRR 1.44 [95% CI 1.43, 1.45]; *p* < 0.001), dihydrocodeine (aIRR 1.25 [95% CI 1.25, 1.26]; *p* < 0.001), oxycodone (aIRR 1.21 [95% CI 1.15, 1.27]; *p* < 0.001), tramadol (aIRR 1.15 [95% CI 1.13, 1.16]; *p* < 0.001), and fentanyl (aIRR 1.06 [95% CI 1.03, 1.10]; *p* < 0.001). There were similar trends in the full unmatched cohort (Table C in S1 Appendix).

**Table 2:** IRR estimates of the association between prenatal opioid exposure and hospital admissions among children using a 1:5 PS-matched cohort (2010–2017).

	Incidence rate (per 1,000 person-years)^*^	Rate difference (per 1,000 person-years)^*^	IRR of admission events (95% CI)		
			Crude^†^	Model 1^a^	Model 2^b^
Opioid exposure during pregnancy					
No	183.3	0 (reference)	1 (reference)	1 (reference)	1 (reference)
Yes	242.4	59.1	**1.32 (1.32, 1.33)**	**1.28 (1.27, 1.28)**	**1.28 (1.28, 1.29)**
Timing of opioid exposure					
No opioid exposure	183.3	0 (reference)	1 (reference)	1 (reference)	1 (reference)
First trimester only	230.8	47.5	**1.26 (1.25, 1.27)**	**1.21 (1.20, 1.22)**	**1.21 (1.21, 1.22)**
Second trimester only	257.9	74.6	**1.41 (1.40, 1.42)**	**1.38 (1.37, 1.39)**	**1.38 (1.37, 1.39)**
Third trimester only	236.4	53.1	**1.29 (1.28, 1.30)**	**1.28 (1.27, 1.29)**	**1.29 (1.28, 1.30)**
More than one trimester	314.3	131.0	**1.71 (1.69, 1.74)**	**1.49 (1.47, 1.51)**	**1.48 (1.46, 1.50)**
Dose-dependent association, MME^c^					
No opioid exposure	183.3	0 (reference)	1 (reference)	1 (reference)	1 (reference)
Low-dose exposure	221.1	37.8	**1.21 (1.20, 1.21)**	**1.18 (1.18, 1.19)**	**1.19 (1.19, 1.20)**
High-dose exposure	270.7	87.4	**1.48 (1.47, 1.49)**	**1.40 (1.39, 1.41)**	**1.40 (1.39, 1.41)**
Days of opioid prescriptions					
0	183.3	0 (reference)	1 (reference)	1 (reference)	1 (reference)
1–7	225.6	42.3	**1.23 (1.22, 1.24)**	**1.21 (1.20, 1.21)**	**1.22 (1.21, 1.22)**
8–11	245.4	62.1	**1.34 (1.33, 1.35)**	**1.30 (1.29, 1.31)**	**1.30 (1.29, 1.31)**
≥12	282.6	99.3	**1.54 (1.53, 1.55)**	**1.43 (1.42, 1.44)**	**1.42 (1.41, 1.43)**
Number of opioid prescriptions					
0–1	183.3	0 (reference)	1 (reference)	1 (reference)	1 (reference)
2	225.0	41.7	**1.23 (1.22, 1.23)**	**1.21 (1.20, 1.21)**	**1.22 (1.21, 1.22)**
≥3	263.8	80.5	**1.44 (1.43, 1.45)**	**1.36 (1.35, 1.37)**	**1.36 (1.35, 1.37)**
Maternal health condition with opioid prescriptions					
No opioid exposure	183.3	0 (reference)	1 (reference)	1 (reference)	1 (reference)
Pain-related^d^	270.2	86.9	**1.47 (1.46, 1.49)**	**1.36 (1.34, 1.38)**	**1.33 (1.32, 1.35)**
Antitussive-related^e^	237.0	53.7	**1.29 (1.29, 1.30)**	**1.25 (1.25, 1.26)**	**1.26 (1.25, 1.27)**
Other	247.0	63.7	**1.35 (1.34, 1.36)**	**1.31 (1.30, 1.32)**	**1.31 (1.31, 1.32)**
Opioid type					
No opioid exposure	183.3	0 (reference)	1 (reference)	1 (reference)	1 (reference)
Codeine	257.8	74.5	**1.41 (1.05, 1.89)**	1.08 (0.81, 1.46)	1.02 (0.76, 1.37)
Dihydrocodeine	233.5	50.2	**1.27 (1.27, 1.28)**	**1.25 (1.24, 1.25)**	**1.25 (1.25, 1.26)**
Fentanyl	204.3	21.0	**1.11 (1.08, 1.15)**	**1.06 (1.03, 1.10)**	**1.06 (1.03, 1.10)**
Morphine	297.5	114.2	**1.62 (1.57, 1.68)**	**1.62 (1.57, 1.68)**	**1.62 (1.57, 1.68)**
Oxycodone	221.0	37.7	**1.21 (1.15, 1.27)**	**1.19 (1.14, 1.25)**	**1.21 (1.15, 1.27)**
Tramadol	215.7	32.4	**1.18 (1.16, 1.19)**	**1.14 (1.13, 1.16)**	**1.15 (1.13, 1.16)**
Polytherapy	286.3	103.0	**1.56 (1.55, 1.57)**	**1.44 (1.43, 1.45)**	**1.44 (1.43, 1.45)**

Numbers in bold indicate significant differences (*p* < 0.05).

*Incidence rates were calculated as the number of hospital admissions per 1,000 person-years. Rate differences represent the absolute difference in incidence rates between the opioid-exposed and unexposed groups per 1,000 person-years.

†Crude estimates were unadjusted.

^a^Model 1 was adjusted for maternal age at delivery; infant sex; region of residence (urban and rural); household income level (1st, 2nd, 3rd, and 4th quartiles); birth season (spring, summer, autumn, and winter); parity (1 and ≥2); SMM score; number of hospital outpatient visits and admissions in the year before pregnancy; preterm birth; low birth weight; delivery type (vaginal delivery and cesarean section); and year of delivery (2010–2012, 2013–2015, and 2016–2017).

^b^Model 2 was adjusted for all variables in Model 1, with further adjustment for maternal history of substance use disorders and neuropsychiatric disorders, and plurality (number of infants per delivery).

^c^Low and high doses were divided based on the median value (18 MME).

^d^Pain-related condition includes medical conditions such as neoplasm (benign), diseases of the blood and blood-forming organs and certain disorders involving the immune mechanism, endocrine, nutritional and metabolic diseases, neuropsychiatric disorders, diseases of the nervous system, diseases of the ear and mastoid process, diseases of the circulatory system, diseases of digestive system, diseases of the skin and subcutaneous tissue, diseases of musculoskeletal system and connective tissue, and diseases of the genitourinary system.

^e^Antitussive-related conditions include medical conditions such as infectious and parasitic diseases, and diseases of the respiratory system.

*Abbreviation:* IRR, incidence rate ratio; CI, confidence interval; MME, morphine milligram equivalent; PS, propensity score; SMM, severe maternal morbidity.

Regardless of the various causes of morbidity, including infection, respiratory non-infection, gastrointestinal non-infection, oral cavity, injury/external, and genitourinary non-infection, all causes were associated with increased rates of hospital admission (Fig 2 and Tables D and E in S1 Appendix). The highest hospital admission rates were observed for respiratory non-infection (aIRR 1.34 [95% CI 1.31, 1.37]; *p* < 0.001), followed by infections (aIRR 1.30 [95% CI 1.29, 1.30]; *p* < 0.001), oral cavity (aIRR 1.30 [95% CI 1.22, 1.40]; *p* < 0.001), gastrointestinal non-infection (aIRR 1.15 [95% CI 1.12, 1.18]; *p* < 0.001), genitourinary non-infection (aIRR 1.11 [95% CI 1.08, 1.14]; *p* < 0.001), and injury/external conditions (aIRR 1.10 [95% CI 1.08, 1.12]; *p* < 0.001) in the PS-matched cohort. The association between prenatal opioid exposure and hospital admissions was up to 22% greater for respiratory non-infection than for other diagnostic categories based on relative aIRR estimates (range, 3%–22%). Fig 3 presents the dose-dependent subgroup analysis of the association between maternal opioid use during pregnancy and the hospital admission rates by causes of morbidity, showing dose-dependent associations particularly for infection, respiratory non-infection, genitourinary non-infection, gastrointestinal non-infection, and injury/external conditions.

**Fig 2:**
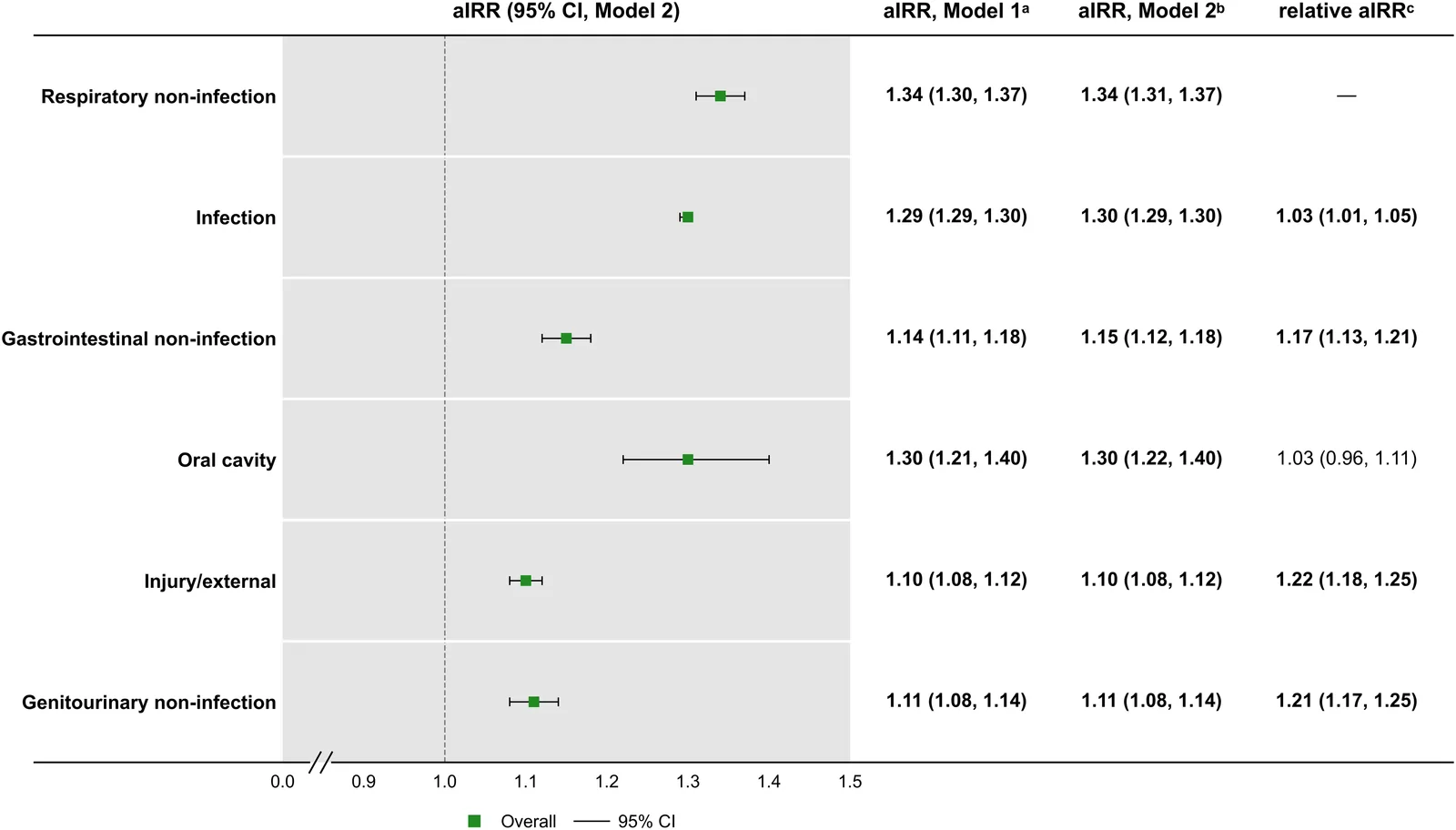
Adjusted incidence rate ratio estimates of the association between prenatal opioid exposure and hospital admissions among children, using a 1:5 PS-matched cohort (2010–2017). *Abbreviation:* aIRR, adjusted incidence rate ratio; CI, confidence interval; SMM, severe maternal morbidity. Numbers in bold indicate significant differences (*p* < 0.05). *P*-values were derived from a generalized linear model with a Poisson distribution and a log link function. ^a^Model 1 was adjusted for maternal age at delivery; infant sex; region of residence (urban and rural); household income level (1st, 2nd, 3rd, and 4th quartiles); birth season (spring, summer, autumn, and winter); parity (1 and ≥2); SMM score; number of hospital outpatient visits and admissions in the year before pregnancy; preterm birth; low birth weight; delivery type (vaginal delivery or cesarean section); and year of delivery (2010–2012, 2013–2015, and 2016–2017). ^b^Model 2 was adjusted for all variables in Model 1, with further adjustment for maternal history of substance use disorders and neuropsychiatric disorders, and plurality (number of infants per delivery). ^c^Relative IRRs represent the comparison of the IRR for respiratory non-infection with the IRR for each other outcome category (infection, gastrointestinal non-infection, oral cavity, injury/external, and genitourinary non-infection).

**Fig 3:**
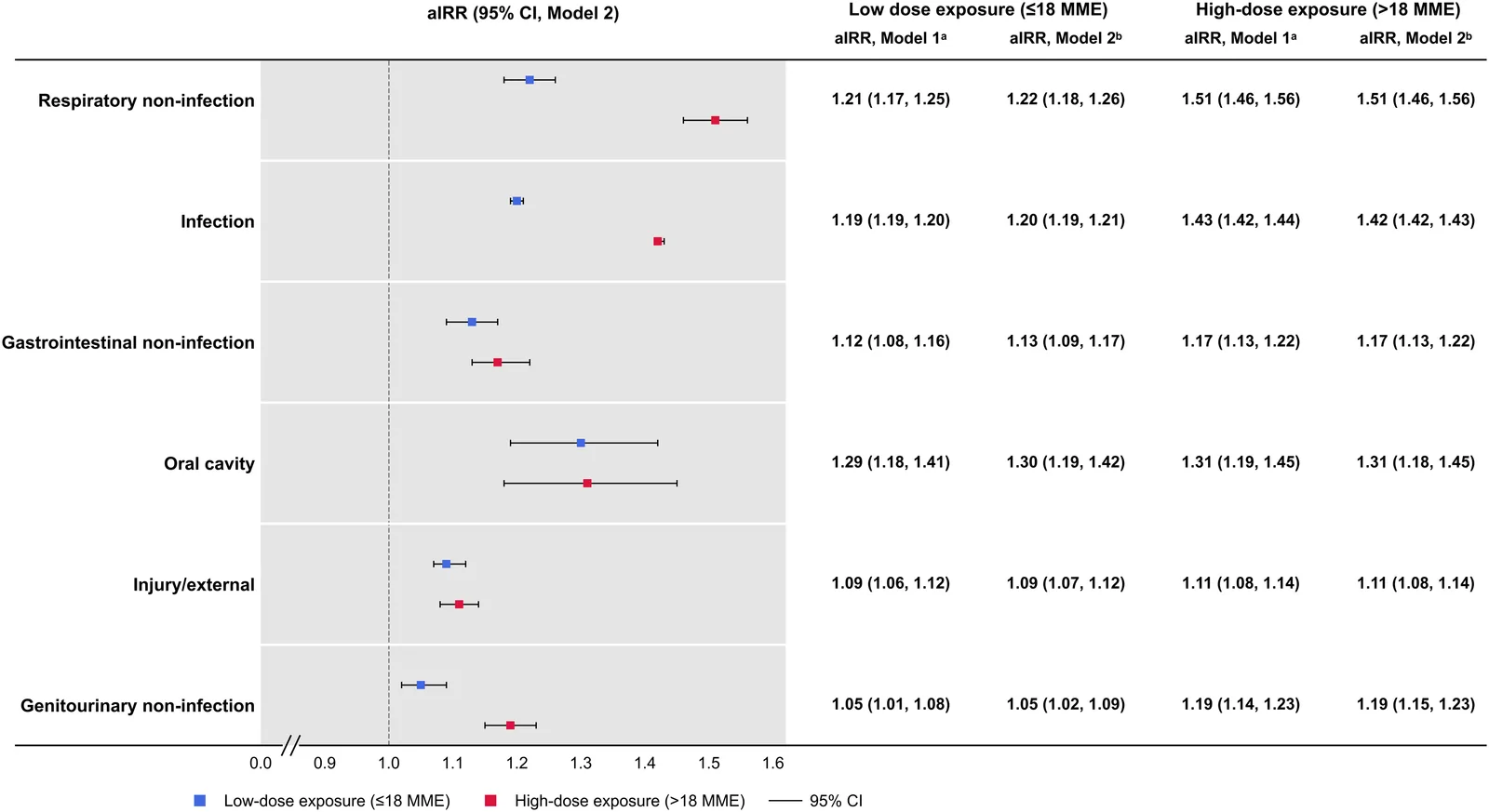
Adjusted incidence rate ratio estimates of the dose-dependent association between prenatal opioid exposure and hospital admissions among children, using a 1:5 PS-matched cohort (2010–2017). *Abbreviation:* aIRR, adjusted incidence rate ratio; CI, confidence interval; MME, morphine milligram equivalent. Numbers in bold indicate significant differences (*p* < 0.05). *P*-values were derived from a generalized linear model with a Poisson distribution and a log link function. Low and high doses were divided based on the median value (18 MME). ^a^Model 1 was adjusted for maternal age at delivery; infant sex; region of residence (urban and rural); household income level (1st, 2nd, 3rd, and 4th quartiles); birth season (spring, summer, autumn, and winter); parity (1 and ≥2); SMM score; number of hospital outpatient visits and admissions in the year before pregnancy; preterm birth; low birth weight; delivery type (vaginal delivery or cesarean section); and year of delivery (2010–2012, 2013–2015, and 2016–2017). ^b^Model 2 was adjusted for all variables in Model 1, with further adjustment for maternal history of substance use disorders and neuropsychiatric disorders, and plurality (number of infants per delivery).

As the children grew older, the association between prenatal opioid exposure and hospital admission gradually attenuated, with aIRRs of 1.19 (95% CI [1.19, 1.20]; *p* < 0.001), 1.08 (95% CI [1.06, 1.09]; *p* < 0.001), and 1.07 (95% CI [1.04, 1.10]; *p* < 0.001) in those aged 0–1, 2–3, and 4–5 years, respectively, compared to non-exposed children (Table 3). Dose-dependent associations were observed in children aged 0–1, 2–3, and 4–5 years, with higher aIRRs for high-dose than for low-dose exposure, but were not evident beyond five years of age.

**Table 3:** IRR estimates of the dose-dependent association between prenatal opioid exposure and hospital admissions among children, stratified by age group using a 1:5 PS-matched cohort (2010–2017).

Age at admission	Overall				Low-dose exposure (≤18 MME)				High-dose exposure (>18 MME)			
	Incidence rate (per 1,000 PY)^*^	Crude^†^	Model 1^a^	Model 2^b^	Incidence rate (per 1,000 PY)^*^	Crude^†^	Model 1^a^	Model 2^b^	Incidence rate (per 1,000 PY)^*^	Crude^†^	Model 1^a^	Model 2^b^
0–1 years												
No opioid exposure	342.5	1 (reference)	1 (reference)	1 (reference)	342.5	1 (reference)	1 (reference)	1 (reference)	342.5	1 (reference)	1 (reference)	1 (reference)
Opioid exposure	414.6	**1.21 (1.21, 1.22)**	**1.19 (1.18, 1.19)**	**1.19 (1.19, 1.20)**	387.4	**1.13 (1.12, 1.14)**	**1.14 (1.14, 1.15)**	**1.15 (1.14, 1.15)**	447.4	**1.31 (1.30, 1.31)**	**1.24 (1.23, 1.25)**	**1.24 (1.23, 1.25)**
2–3 years												
No opioid exposure	179.6	1 (reference)	1 (reference)	1 (reference)	179.6	1 (reference)	1 (reference)	1 (reference)	179.6	1 (reference)	1 (reference)	1 (reference)
Opioid exposure	195.1	**1.09 (1.07, 1.10)**	**1.07 (1.06, 1.09)**	**1.08 (1.06, 1.09)**	190.6	**1.06 (1.04, 1.08)**	**1.06 (1.04, 1.08)**	**1.06 (1.04, 1.08)**	201.8	**1.12 (1.10, 1.15)**	**1.10 (1.08, 1.12)**	**1.10 (1.08, 1.13)**
4–5 years												
No opioid exposure	110.2	1 (reference)	1 (reference)	1 (reference)	110.2	1 (reference)	1 (reference)	1 (reference)	110.2	1 (reference)	1 (reference)	1 (reference)
Opioid exposure	119.2	**1.08 (1.05, 1.11)**	**1.07 (1.04, 1.10)**	**1.07 (1.04, 1.10)**	115.6	**1.05 (1.01, 1.08)**	**1.04 (1.01, 1.08)**	**1.04 (1.01, 1.08)**	124.6	**1.13 (1.09, 1.17)**	**1.11 (1.07, 1.15)**	**1.11 (1.07, 1.15)**
6–7 years												
No opioid exposure	23.9	1 (reference)	1 (reference)	1 (reference)	23.9	1 (reference)	1 (reference)	1 (reference)	23.9	1 (reference)	1 (reference)	1 (reference)
Opioid exposure	25.9	**1.08 (1.04, 1.13)**	1.01 (0.97, 1.05)	1.01 (0.97, 1.05)	31.2	**1.31 (1.24, 1.37)**	1.01 (0.96, 1.06)	1.01 (0.96, 1.07)	20.7	**0.87 (0.81, 0.92)**	1.01 (0.95, 1.08)	1.01 (0.95, 1.08)
8–10 years												
No opioid exposure	9.3	1 (reference)	1 (reference)	1 (reference)	9.3	1 (reference)	1 (reference)	1 (reference)	9.3	1 (reference)	1 (reference)	1 (reference)
Opioid exposure	10.6	**1.14 (1.08, 1.21)**	1.05 (0.99, 1.11)	1.05 (0.99, 1.12)	10.4	**1.12 (1.04, 1.20)**	1.02 (0.95, 1.09)	1.02 (0.95, 1.10)	11.0	**1.19 (1.09, 1.30)**	**1.10 (1.01, 1.20)**	**1.11 (1.02, 1.21)**
11–14 years												
No opioid exposure	2.7	1 (reference)	1 (reference)	1 (reference)	2.7	1 (reference)	1 (reference)	1 (reference)	2.7	1 (reference)	1 (reference)	1 (reference)
Opioid exposure	2.9	1.09 (0.98, 1.21)	1.09 (0.99, 1.22)	1.10 (0.99, 1.22)	3.1	**1.14 (1.01, 1.29)**	1.14 (1.00, 1.29)	1.15 (1.00, 1.30)	2.7	1.00 (0.84, 1.19)	1.01 (0.85, 1.19)	1.01 (0.85, 1.20)

Numbers in bold indicate significant differences (*p* < 0.05).

Low and high doses were divided based on the median value (18 MME).

*Incidence rates were calculated as the number of hospital admissions per 1,000 person-years.

†Crude estimates were unadjusted.

^a^Model 1 was adjusted for maternal age at delivery; infant sex; region of residence (urban and rural); household income level (1st, 2nd, 3rd, and 4th quartiles); birth season (spring, summer, autumn, and winter); parity (1 and ≥2); SMM score; number of hospital outpatient visits and admissions in the year before pregnancy; preterm birth; low birth weight; delivery type (vaginal delivery and cesarean section); and year of delivery (2010–2012, 2013–2015, and 2016–2017).

^b^Model 2 was adjusted for all variables in Model 1, with further adjustment for maternal history of substance use disorders and neuropsychiatric disorders, and plurality (number of infants per delivery).

*Abbreviation:* IRR, incidence rate ratio; CI, confidence interval; MME, morphine milligram equivalent; PS, propensity score; PY, person-year; SMM, severe maternal morbidity.

The sibling comparison analysis showed attenuated aIRR estimates, indicating the potential influence of shared familial factors, but the association between prenatal opioid exposure and hospital admission rates remained statistically significant (aIRR 1.08 [95% CI 1.07, 1.10]; *p* < 0.001; Table 4). In the sibling cohort analysis, a similar dose-dependent pattern was observed, consistent with findings from the PS-matched cohort (high-dose exposure: aIRR 1.19 [95% CI 1.17, 1.20]; *p* < 0.001). Consistent with this, analyses in the PS-matched sibling comparison cohort and models excluding potential mediators from the adjustment variables also indicated higher hospital admission rates among children with prenatal opioid exposure compared with non-exposed children (Tables F–H in S1 Appendix). In a sensitivity analysis, the association was also robust to the exclusion of pregnancies with a single opioid prescription from the reference group, and was stronger in the full unmatched cohort than in the primary analysis (aIRR 1.41 [95% CI 1.41, 1.42]; *p* < 0.001), while remaining significant in the re-matched PS cohort (aIRR 1.25 [95% CI 1.24, 1.26]; *p* < 0.001) and the re-derived sibling comparison cohort (aIRR 1.05 [95% CI 1.02, 1.07]; *p* < 0.001; Table I in S1 Appendix). Similar effect sizes and patterns of hospital admission rates were found in all different groups following stratification by several maternal and infant characteristics, including maternal age at delivery year, region of residence, income levels, number of parities, delivery type, infant sexes, and birth seasons (Table J in S1 Appendix).

**Table 4:** IRR estimates of the association between prenatal opioid exposure and hospital admissions among children using a sibling comparison cohort (2010–2017).

	Incidence rate (per 1,000 person-years)^*^	Rate difference per (1,000 person-years)^*^	IRR of admission events (95% CI)		
			Crude^†^	Model 1^a^	Model 2^b^
Opioid exposure during pregnancy					
No	216.9	Reference	1 (reference)	1 (reference)	1 (reference)
Yes	233.9	17.0	**1.20 (1.17, 1.22)**	**1.09 (1.07, 1.10)**	**1.08 (1.07, 1.10)**
Timing of opioid exposure					
No opioid exposure	216.9	Reference	1 (reference)	1 (reference)	1 (reference)
First trimester only	219.3	2.4	**1.12 (1.09, 1.16)**	**1.02 (1.00, 1.03)**	**1.02 (1.00, 1.03)**
Second trimester only	250.6	33.7	**1.26 (1.20, 1.31)**	**1.17 (1.14, 1.19)**	**1.16 (1.14, 1.18)**
Third trimester only	230.6	13.7	**1.17 (1.13, 1.22)**	**1.08 (1.06, 1.10)**	**1.08 (1.06, 1.10)**
More than one trimester	302.2	85.3	**1.55 (1.45, 1.66)**	**1.37 (1.33, 1.41)**	**1.34 (1.31, 1.38)**
Dose-dependent association, MME^c^					
No opioid exposure	216.9	Reference	1 (reference)	1 (reference)	1 (reference)
Low-dose exposure	212.7	−4.2	**1.08 (1.04, 1.11)**	1.00 (0.98, 1.01)	1.00 (0.98, 1.01)
High-dose exposure	259.7	42.8	**1.32 (1.28, 1.35)**	**1.19 (1.18, 1.21)**	**1.19 (1.17, 1.20)**
Days of opioid prescriptions					
0	216.9	Reference	1 (reference)	1 (reference)	1 (reference)
1–7	217.4	0.5	**1.10 (1.07, 1.13)**	**1.02 (1.00, 1.03)**	**1.02 (1.00, 1.03)**
8–11	236.8	19.9	**1.18 (1.13, 1.23)**	**1.10 (1.08, 1.12)**	**1.09 (1.07, 1.12)**
≥12	270.0	53.1	**1.40 (1.35, 1.45)**	**1.25 (1.22, 1.27)**	**1.23 (1.21, 1.25)**
Number of opioid prescriptions					
0–1	216.9	Reference	1 (reference)	1 (reference)	1 (reference)
2	215.8	−1.1	**1.09 (1.06, 1.13)**	1.01 (0.99, 1.03)	1.01 (1.00, 1.03)
≥3	254.8	37.9	**1.30 (1.27, 1.34)**	**1.17 (1.16, 1.19)**	**1.16 (1.15, 1.18)**
Maternal health condition with opioid prescriptions					
No opioid exposure	216.9	Reference	1 (reference)	1 (reference)	1 (reference)
Pain-related^d^	243.9	27.0	**1.29 (1.20, 1.38)**	**1.10 (1.07, 1.13)**	**1.08 (1.05, 1.11)**
Antitussive-related^e^	232.0	15.1	**1.19 (1.16, 1.22)**	**1.08 (1.06, 1.09)**	**1.07 (1.06, 1.09)**
Other	235.4	18.5	**1.20 (1.15, 1.24)**	**1.10 (1.08, 1.13)**	**1.10 (1.08, 1.12)**
Opioid type					
No opioid exposure	216.9	Reference	1 (reference)	1 (reference)	1 (reference)
Codeine	32.3	−184.6	0.14 (0.00, 792.33)	0.06 (0.00, 6.75)	0.05 (0.00, 6.16)
Dihydrocodeine	227.0	10.1	**1.14 (1.11, 1.17)**	**1.05 (1.04, 1.07)**	**1.05 (1.04, 1.07)**
Fentanyl	192.4	−24.5	0.95 (0.77, 1.17)	**0.86 (0.80, 0.92)**	**0.86 (0.80, 0.92)**
Morphine	250.7	33.8	**1.20 (1.03, 1.39)**	**1.15 (1.09, 1.22)**	**1.15 (1.09, 1.22)**
Oxycodone	229.2	12.3	1.18 (0.85, 1.62)	**1.12 (1.00, 1.24)**	**1.12 (1.01, 1.25)**
Tramadol	205.3	−11.6	**1.11 (1.03, 1.20)**	1.00 (0.97, 1.03)	1.00 (0.97, 1.03)
Polytherapy	270.6	53.7	**1.40 (1.35, 1.46)**	**1.24 (1.21, 1.26)**	**1.22 (1.20, 1.25)**

Numbers in bold indicate significant differences (*p* < 0.05).

*Incidence rates were calculated as the number of hospital admissions per 1,000 person-years. Rate differences represent the absolute difference in incidence rates between the opioid-exposed and unexposed groups per 1,000 person-years.

†Crude estimates were obtained from conditional Poisson models stratified on family, including prenatal opioid exposure and the person-year offset only, without further covariate adjustment.

^a^Model 1 was adjusted for maternal age at delivery; infant sex; region of residence (urban and rural); household income level (1st, 2nd, 3rd, and 4th quartiles); birth season (spring, summer, autumn, and winter); parity (1 and ≥2); SMM score; number of hospital outpatient visits and admissions in the year before pregnancy; preterm birth; low birth weight; delivery type (vaginal delivery and cesarean section); and year of delivery (2010–2012, 2013–2015, and 2016–2017).

^b^Model 2 was adjusted for all variables in Model 1, with further adjustment for maternal history of substance use disorders and neuropsychiatric disorders, and plurality (number of infants per delivery).

^c^Low and high doses were divided based on the median value (18 MME).

^d^Pain-related condition includes medical conditions such as neoplasm (benign), diseases of the blood and blood-forming organs and certain disorders involving the immune mechanism, endocrine, nutritional and metabolic diseases, neuropsychiatric disorders, diseases of the nervous system, diseases of the ear and mastoid process, diseases of the circulatory system, diseases of digestive system, diseases of the skin and subcutaneous tissue, diseases of musculoskeletal system and connective tissue, and diseases of the genitourinary system.

^e^Antitussive-related conditions include medical conditions such as infectious and parasitic diseases, and diseases of the respiratory system.

*Abbreviation:* IRR, incidence rate ratio; CI, confidence interval; MME, morphine milligram equivalent; SMM, severe maternal morbidity.

## Discussion

We investigated the association between prenatal opioid exposure and subsequent hospital admissions in children and obtained several key findings. First, in this nationwide population-based cohort study that included 118,671 infants exposed to opioids among 2,928,186 infants, prenatal exposures to opioids were significantly associated with an increased risk for subsequent hospital admissions in children with 28% increase. The sibling-controlled analyses showed an attenuated association, with a modest increase in hospital admission among children with maternal opioid prescriptions, indicating that the potential influence of shared familial factors may explain part of the observed association. Second, the rates of hospital admission in children showed a dose-dependent manner with maternal opioid dose and duration of opioid prescriptions. Third, when children grew older, the hospital admission rates decreased with time-attenuating relationships but remained evident up to five years of age. Fourth, pain-related reasons for maternal opioid prescriptions showed slightly higher rates of hospital admission in their children compared to antitussive-related prescriptions of opioids during pregnancy. Finally, children with prenatal opioid exposure showed increased hospital admissions, regardless of the causes of morbidity. Compared to non-exposed children, those with prenatal opioid exposure had significantly higher rates of hospital admissions for several causes of morbidities, including infections, respiratory non-infections, oral cavity, gastrointestinal non-infections, genitourinary non-infections, and injury/external conditions.

Previous studies have examined the association between maternal opioid use and various health outcomes in the offspring [3,10,29–31]. While some studies explored associations of opioid use with antenatal and postnatal admissions or SMM in mothers [32,33], studies focusing on associations between maternal opioid use and subsequent risks of hospital admissions in their children remain limited. Although a few studies have explored associations between maternal opioid treatment after delivery and adverse infant outcomes, these had limited cohort sizes (*n* = 13,320 or 865,691 mother–infant pairs) or did not specifically examine opioid use during pregnancy [11,12]. Moreover, the study designs of previous studies were not robust enough to characterize the associations between prenatal opioid exposure and dose-dependent and/or time-attenuated relationships stratified by cause of morbidity and failed to adequately account for potential confounders, including maternal and childbirth-related factors. Notably, sibling comparison analysis, which helps control for shared familial and genetic factors, was rarely employed. By contrast, our large-scale nationwide cohort study, encompassing over 2.92 million children, offers additional evidence supported by statistical analyses, including sibling comparison analysis, thereby providing further evidence of the association between prenatal opioid exposure and increased hospital admission rates in children.

This study suggested that prenatal opioid exposure is associated with an increased risk of hospital admissions in children, potentially through several underlying biological pathways. Opioids are known to cross the placenta, and in utero opioid exposure may directly impair fetal immune development and overall health resilience [34]. Prior studies have reported that neonates with prenatal opioid exposures had lower neutrophil counts, reduced inflammatory cytokine levels, and decreased IL-2 production during CD4+ T cell proliferations, implying dysregulated immune systems from birth [35]. In addition, opioids possess immunosuppressive properties (particularly morphine and codeine), which have been related to increased susceptibility to infections, including pneumonia [9]. Our findings also showed similar results with this potential mechanism, as hospital admissions due to infections were substantially higher among children with prenatal opioid exposure. Furthermore, opioids could disrupt bone metabolism through inhibition of osteoblast differentiation, suppression of bone formation, and reduction in bone mineral density [36,37], potentially increasing susceptibility to fractures and thereby contributing to the elevated risk of hospital admissions due to injury.

The observed dose-dependent manner in our findings supported that higher cumulative dose and longer duration of prescriptions were associated with greater rates of hospital admission, consistent with a potential biological gradient [1]. However, this dose-response relationship was not uniform across outcome categories. A clear dose-dependent gradient was observed for infection-related and non-infection respiratory admissions, whereas estimates for gastrointestinal, oral cavity, injury/external, and genitourinary admissions were broadly similar across low- and high-dose groups, suggesting that the dose-response signal is largely driven by infection-related outcomes. Notably, as infection-related admissions represented the predominant cause of hospitalization in this cohort, the overall “any hospitalization” endpoint was likewise largely driven by this outcome. Mothers prescribed opioids for pain, often involving higher doses or prolonged use, had children with higher hospital admission rates compared to those prescribed antitussive opioids [8]. Importantly, high-dose opioid prescriptions may also be indicative of more severe underlying maternal conditions, which themselves could influence child outcomes.

Moreover, the association between prenatal opioid exposure and hospital admission was notably strongest in the first year of life and attenuated with increasing age, underscoring that the critical window of vulnerability is concentrated in early infancy. This temporal pattern highlights the particular importance of vigilant postnatal monitoring and early intervention during this period. The significant increase in overall hospital admission rates among children with prenatal opioid exposure underscores the need for targeted postnatal care and routine child health screening, especially within the first year of life. Early identification of at-risk children and timely intervention may substantially mitigate the morbidity burden associated with maternal opioid use during pregnancy.

Subsequent or long-term outcomes of children born to individuals with maternal opioid prescriptions have not been well characterized. In this cohort study, we observed that children with prenatal opioid exposure had a higher rate of hospital admissions compared with children without such exposure. Although the association was attenuated when comparing siblings with different exposure statuses, suggesting that familial factors may partly explain the observed association, these findings highlight the importance of supporting and monitoring pregnant women with opioid prescriptions prenatally and postnatally, as well as their children, to mitigate subsequent adverse outcomes. In cases where pregnant individuals experience severe conditions such as chronic pain, avoiding opioid prescriptions may not be feasible, as opioid treatment can provide meaningful symptom relief and functional improvement, potentially resulting in maternal and neonatal health benefits [8,10]. However, opioids should be used during pregnancy when the anticipated benefits clearly outweigh the potential risks.

Particular caution is warranted with high-dose and long-term opioid prescriptions during pregnancy. Before initiating opioid prescriptions, clinicians should engage in shared decision-making with pregnant women to establish clear treatment goals and consider discontinuation if treatment goals are not met or if risks outweigh benefits [8]. Continuous monitoring of the risks and benefits of opioid prescriptions should occur at least every three months, or more frequently, and prescription data should be reviewed through prescription drug monitoring programs and routine health checks for pregnant women. For pregnant individuals with opioid use disorders, opioid agonist pharmacotherapy, including medications such as buprenorphine or methadone, should be initiated or adjusted as appropriate [38]. In addition, regular child screening checks and postnatal follow-up for children born to mothers with opioid prescriptions during pregnancy are imperative to identify and address potential health challenges early.

This study has several limitations. First, although this nationwide longitudinal birth cohort study involved validated diagnostic records and a large generalizable sample size, the potential risk of selection and recall biases was not fully avoided. Second, identification of exposure was conducted based on prescription records, which may not fully capture actual medication intake, potentially resulting in exposure misclassification. However, as opioids are only legally available by prescription in South Korea, where illicit opioid use remains exceptionally low compared with other countries, misclassification attributable to non-prescribed or over-the-counter use is unlikely to have meaningfully affected our results [3,39]. Nevertheless, because clinically meaningful prenatal opioid exposure was defined as two or more opioid prescriptions within a trimester, pregnancies with a single prescription were included in the reference group. In a sensitivity analysis excluding these pregnancies, the estimated associations were similar or stronger than in the primary analysis, suggesting that our estimates were, if anything, conservative. Third, the observational study design limits causal inference, and findings should be interpreted as associations rather than a direct causal relationship. Fourth, this study focused exclusively on hospital admissions to reflect clinically important illnesses, as conditions typically managed in primary care or outpatient settings were not included. Fifth, only the primary diagnosis was considered for each hospital admission. As a result, secondary diagnoses of morbidities, such as infections, non-infectious respiratory conditions, non-infectious gastrointestinal conditions, oral cavity diseases, injuries/external causes, and non-infectious genitourinary conditions could have overlapped within the same admission. However, duplicate cases with the same admission date of primary diagnosis were excluded, and the corresponding admissions were counted only once. Sixth, although sibling-controlled analyses help adjust for shared familial and genetic factors, families with discordant exposure status may differ from the general population, and residual confounding by unobserved non-shared confounders may still be present [40,41]. Seventh, despite a follow-up period of up to 14 years, the sample size in later childhood (11–14 years) was relatively limited. In addition, the data may not fully capture developmental or environmental influences that emerge in late childhood. Therefore, findings for these age groups should be interpreted with caution. Eighth, despite the use of a previously validated algorithm to estimate pregnancy onset, some misclassification of the exposure window may persist [40]. Ninth, other potential teratogenic medications were not considered in this analysis, which may have resulted in residual confounding. Lastly, the study was restricted to pregnancies resulting in live births, as gestational age data were not available for terminated pregnancies, potentially introducing selection bias.

Taken together, maternal opioid use during pregnancy was significantly associated with the subsequent risk of hospital admission in children, with a dose-dependent relationship and time-attenuation effects. These significant associations were shown across several causes of morbidities, including infections, respiratory non-infections, oral cavity, gastrointestinal non-infections, genitourinary non-infections, and injury/external conditions. Although the association persisted in the sibling comparison cohort, the attenuated estimate suggested that shared familial factors may partly explain the observed association. These findings warrant profound caution for the need for routine child screening and postnatal health checks, especially for those born to mothers with opioid prescriptions during pregnancy. Further, clinicians should be aware of the potential for an increase in the risk of hospital admission in children following maternal opioid prescriptions and counsel pregnant women about this risk.

### Ethical approval

The Korean government anonymized all patient-related data, including personal identification numbers, to enhance confidentiality. This study was approved by the Institutional Review Board of Kyung Hee University (KHSIRB-24–511[EA]), which waived the requirement for informed consent because the study used de-identified administrative data, and access to the data was obtained from the Korean NHIS (NHIS-2025-03-1-040). We conducted this study using de-identified administrative data that were obtained without prior consent.

### Transparency

The lead authors (H.J.K., Y.Y., J.K. and D.K.Y.) affirm that the manuscript is an honest, accurate, and transparent account of the study being reported; that no important aspects of the study have been omitted; and that any discrepancies from the study as planned (and, if relevant, registered) have been explained.

### Dissemination to participants and related patient and public communities

The research questions and outcome measures were independently determined without the involvement of the children or their parents. The study design and implementation were conducted without consultation. However, we plan to disseminate the results of this study to all study participants and wider relevant communities upon request.

## Supporting information

S1 Appendix Supplementary Fig A and Tables A–J. (DOCX)

S1 Checklist The RECORD statement—checklist of items, extended from the STROBE statement, that should be reported in observational studies using routinely collected health data. (DOCX)

## Abbreviations

aIRR: Adjusted incidence rate ratio
CIs: confidence intervals
ICD: International Classification of Diseases
IRR: incidence rate ratio
MME: morphine milligram equivalents
NHIS: National Health Insurance Service
PS: propensity score
SMD: standardized mean difference
SMM: severe maternal morbidity
