# Supplementary material for: Maternal opioid prescriptions and subsequent risk of hospital admission in offspring in South Korea: A nationwide birth cohort study
Source: PLoS Med. 2026 Oct 13;23(10):e1004829. doi: 10.1371/journal.pmed.1004829 (PMC13660889; doi:10.1371/journal.pmed.1004829)
Supplement: Supplementary file 1 [file pmed.1004829.s001.docx]

**S1 Appendix**

Original article

**Maternal opioid prescriptions and subsequent risk of hospital admission in offspring in South Korea: A nationwide birth cohort study**

Subtitle: **Maternal opioid prescriptions and childhood hospital admission, a retrospective study**

Hyeon Jin Kim,^1,2¶^ Yesol Yim,^1,2¶^ Christa J Nehs,^3,4^ Jaeyu Park,^1,2^ Eun Soo Kim,^5^ Sung Joon Cho,^5^ Guillaume Fond,^6^ [Laurent Boyer](https://pubmed.ncbi.nlm.nih.gov/?sort=date&size=200&term=Boyer+L&cauthor_id=40680830),^6^ Jiseung Kang,^7,8*^ Dong Keon Yon^1,2,9*^

^¶^ HJK and YY are joint first authors.

* Corresponding authors:

**Dong Keon Yon,** MD, PhD, FACAAI, FAAAAI, ATSF

Department of Pediatrics, Kyung Hee University College of Medicine, 23 Kyungheedae-ro, Dongdaemun-gu, Seoul, 02447, South Korea

Email: [yonkkang@gmail.com](mailto:yonkkang@gmail.com)

**Jiseung Kang**, PhD

School of Health and Environmental Science, Korea University College of Health Science, Anam-ro 145, Seongbuk-gu, Seoul 02841, South Korea

E-mail: wltmd1006@gmail.com

**Contents**

**Supplementary Materials ……………….…………………………………….…….………3**

**Fig A.** Density plot of 1:5 propensity scores of children with and without opioid exposure from 2010 to 2017.

**Table A.** Variable ICD codes used to define exclusion criteria, exposure, outcome diagnosis, and covariates.

**Table B.** Baseline characteristics of participants in the full unmatched cohort and the sibling comparison cohort,2010-2017.

**Table C.** IRR estimates of the association between prenatal opioid exposure and hospital admissions among children using a full unmatched cohort (2010-2017).

**Table D.** IRR estimates of the dose-dependent association between prenatal opioid exposure and hospital admissions among children, using a full unmatched cohort (2010-2017).

**Table E.** IRR estimates of the dose-dependent association between prenatal opioid exposure and hospital admissions among children, using a sibling comparison cohort (2010-2017).

**Table F.** Baseline characteristics of participants in the PS-matched sibling comparison cohort, 2010-2017.

**Table G.** IRR estimates of the association between prenatal opioid exposure and hospital admissions among children using a PS-matched sibling comparison cohort (2010-2017).

**Table H.** Sensitivity analysis excluding potential mediators from the association between prenatal opioid exposure and hospital admissions among children using a 1:5 PS-matched cohort and sibling comparison cohort (2010-2017).

**Table I.** Sensitivity analysis of the association between prenatal opioid exposure and hospital admissions among children after excluding pregnancies with a single opioid prescription from the reference group (2010–2017).

**Table J.** Stratified IRR estimates of the association between prenatal opioid exposure and hospital admissions among children using a 1:5 PS-matched cohort (2010-2017).

**End ……………………………………………………………………………….……….…40**

**Fig A.** Density plot of 1:5 propensity scores of children with and without opioid exposure from 2010 to 2017.

**Table A.** Variable ICD codes used to define exclusion criteria, exposure, outcome diagnosis, and covariates.

| **Variable** | **ICD-10 Code** |
| --- | --- |
| **Exclusion criteria** | |
| immunodeficiency disorders | D80-D84, D89 |
| Cystic fibrosis | E84 |
| Chronic kidney disease | N18 |
| Beta-thalassemia or sickle-cell disorders | D56.1, D57 |
| Cancer | C00-C99 |
| **Exposure (maternal health indications related to opioid prescriptions)** | |
| Infectious and parasitic diseases | A00-A99, B00-B99 |
| Neoplasm (benign), Diseases of the blood and blood-forming organs and certain disorders involving the immune mechanism | D00-D99 |
| Endocrine, nutritional and metabolic diseases | E00-E99 |
| Neuropsychiatric disorders | F00-F99 |
| Diseases of the nervous system | G00-G99 |
| Diseases of the ear and mastoid process | H00-H95 |
| Diseases of the circulatory system | I00-I99 |
| Diseases of the respiratory system | J00-J99 |
| Diseases of digestive system | K00-K99 |
| Diseases of the skin and subcutaneous tissue | L00-L99 |
| Diseases of musculoskeletal system and connective tissue | M00-M99 |
| Diseases of the genitourinary system | N00-N99 |
| Other | O00-O99, P00-P99, Q00-Q99, R00-R99, S00-S99, T00-T99, Z00-Z99 |
| **Outcome** | |
| Infection | A00.0-A00.1, A00.9, A01.0-A01.4, A02.0-A02.2, A02.8-A02.9, A03.0-A03.3, A03.8-A03.9, A04.0-A04.9, A05.0-A05.4, A05.8-A05.9, A06.0-A06.5, A06.9, A07.0-A07.3, A07.8-A07.9, A08.0-A08.5, A09-A09.0, A09.9, A15.0-A15.9, A16.0-A16.5, A16.7-A16.9, A17.0-A17.1, A17.8-A17.9, A18.0, A19.0-A19.2, A19.8-A19.9, A20.2-A20.3, A20.7, A21.2-A21.3, A21.7, A22.1-A22.2, A22.7, A23.0-A23.3, A23.8-A23.9, A24.0-A24.1, A25.0-A25.1, A25.9, A31.0-A31.1, A32.1, A32.7, A36.0-A36.2, A37.0-A37.1, A37.8-A37.9, A39-A39.5, A39.8-A39.9, A40.0-A40.3, A40.8-A40.9, A41, A41, A41-A41.4, A41.8-A41.9, A42.0, A44.0, A46, A48.1, A48.3, A51.0-A51.5, A51.9, A52.0-A52.1, A52.1-A52.3, A52.7, A52.7-A52.9, A53.0, A53.9, A54.0-A54.3, A54.3-A54.6, A54.8, A54.8-A54.9, A55, A56.0-A56.4, A56.8, A57, A58, A59.0, A59.0, A60.0-A60.1, A60.9, A63.0, A63.8, A64, A65, A70, A70, A74.0, A74.8, A78, A79.0-A79.1, A79.8-A79.9, A80.1-A80.4, A80.9, A81.8-A81.9, A82.0-A82.1, A82.9, A83.0-A83.6, A83.8-A83.9, A84.0-A84.1, A84.8-A84.9, A85.0-A85.2, A85.8, A86, A87.0-A87.2, A87.8-A87.9, A88.0, A88.8, A89, A90, A91, A92.0-A92.4, A92.8-A92.9, A93.0, A93.2, A93.8, A94, A95.0-A95.1, A95.9, A96.0-A96.2, A96.8-A96.9, A98.0-A98.5, A98.8, A99, B00.0-B00.5, B00.7-B00.9, B01.0-B01.2, B01.8-B01.9, B02.0-B02.3, B02.7-B02.9, B03, B04, B05.0-B05.4, B05.8-B05.9, B06.0, B06.8-B06.9, B07, B08.0-B08.5, B08.8, B09, B15.0, B15.9, B16.0-B16.2, B16.9, B17.0-B17.2, B17.8-B17.9, B18.0-B18.2, B18.8-B18.9, B19.0, B19.9, B20.0-B20.9, B21.0-B21.3, B21.7-B21.9, B22.0-B22.2, B22.7, B23.0-B23.2, B23.8, B24, B25.0-B25.2, B25.8-B25.9, B26.0-B26.3, B26.8, B26.8-B26.9, B27.0-B27.1, B27.8-B27.9, B30.0-B30.3, B30.8-B30.9, B33.0-B33.4, B33.8, B34.0-B34.4, B34.8-B34.9, B35.0-B35.6, B35.8-B35.9, B36.0-B36.3, B36.8, B37, B37.1-B37.4, B38.0-B38.2, B39.0-B39.5, B39.9, B40.0-B40.3, B40.7-B40.8, B41.0, B42.0, B44.0-B44.1, B45.0, B58.3, B59, B71.0, B71.9, B76.0, B78.9, B81.0, B85.0-B85.4, B86, B87.9, B88.0-B88.3, B88.8-B88.9, B95.1, B96, B97.0-B97.8, G00.0-G00.1, G00.1-G00.3, G00.8-G00.9, G01, G02.0, G03.1, G05.0-G05.1, G06.0-G06.2, G07, H19.1, H60.3, H65.0-H65.4, H65.9, H66.0-H66.4, H66.9, H67.0, H67.8, H68.0, H70.0-H70.2, H70.8-H70.9, H72.0-H72.2, H72.8-H72.9, H73.0, H75.0, H83.0, H92.1, I30.1, I32.0, I33.0, I41.0-I41.1, J00, J01.0-J01.4, J01.8-J01.9, J02.0, J02.8-J02.9, J03.0, J03.8-J03.9, J04.0-J04.2, J05.0-J05.1, J06.0, J06.8-J06.9, J09, J10.0-J10.1, J10.8, J11-J11.1, J11.8, J12.0-J12.3, J12.8-J12.9, J13, J14, J15.0-J15.9, J16.0, J16.8, J17.0-J17.3, J17.8, J18.0-J18.1, J18.8-J18.9, J20.0-J20.9, J21.0-J21.1, J21.8-J21.9, J22-J22.0, J32.0-J32.4, J32.8-J32.9, J34.0, J35.0, J36, J39.0-J39.1, J40, J41.0-J41.1, J41.8, J42, J44.0, J47, J65, J85.0-J85.3, J86.0, J86.9, K52.8, L00, L01.0, L02.0-L02.4, L02.8-L02.9, L03, L03.2-L03.3, L03.8-L03.9, L05.0, L05.9, L08.0-L08.1, L08.8-L08.9, L13.0, L30.3, L88, M00, M01, M01.4, M46.2-M46.5, M49.0-M49.3, M60, M63.0-M63.2, M65.0-M65.1, M68.0, M71.0-M71.1, M72.6, M73.0-M73.1, M86-M86.9, M90.0, N13.6, N15.1, N30.0, N30.8, N34.0, N35.1, N39.0, N41.0, N41.2, N43.1, N45.0, N45.9, N51.2, N70.0, N73.0-N73.4, N73.9, N74.2-N74.4, N75.0-N75.1, N76.0, N76.4, O03.0, O03.5, O08.0, O26.4, O86.2, O91, O98.1-O98.2, O98.4-O98.5, P23.0, P23.2-P23.6, P23.8-P23.9, P35.0-P35.3, P35.8-P35.9, P36.0-P36.5, P36.8-P36.9, P37.0, P37.2, P39.0, P39.3-P39.4, Z21 |
| Respiratory non-infection | J30.0-J30.9, J31.0-J31.2, J32.0-J32.9, J33.0-J33.9, J34.0-J34.9, J35.0-J35.9, J36, J37.0-J37.1, J38.0-J38.7, J39.0-J39.9, J40, J41, J42, J43, J44, J45, J47, J80, J81, J82, J84, J85, J86, J90, J91, J92, J93, J94, J95, J96, J98, J99 |
| Gastrointestinal non-infection | K20-K23, K25-K31, K40-K46, K50-K52, K55-K63, K65-K66, K90-K92 |
| Oral cavity | K00-K09.9, K11-K11.9, K12-K12.39, K13-K13.79, K14-K14.9, R13.10, Z012, Z463, Z464 |
| Injury/external | J60-J70, P10, P15, S00, S09-S10, S19-S20, S29-S30, S39-S40, S49-S50, S59-S60, S69-S70, S79-S80, S89-S90, S99, T00, T07-T08, T14-T15, T19-T20, T32-T33, T35-T36, T50-T51, T65-T66, T78-T80, T88, T90, T98, V01, V99, W00, X59, Y85, Y89 |
| Genitourinary non-infection | N00-N08, N10-N23, N25-N37, N39-N51, N60-N64, N70-N77, N80-N94 |
| Appendicitis | K35-K37 |
| **Maternal characteristics** | |
| Severe maternal morbidity^*^ | Pre-existing hypertensive heart disease: O10.1 Pre-existing hypertensive heart and renal disease: O10.3 Eclampsia: O15 Cerebral venous thrombosis in pregnancy: O22.5 Cerebral venous thrombosis in the puerperium: O87.3 Pulmonary, cardiac, and central nervous system complications of anesthesia during pregnancy, labor and delivery, and the puerperium: O29.0, O29.1, O29.2, O89.0, O89.1, O89.2, O74.0, O74.1, O74.2, O74.3 Placental abruption with coagulation defect: O45.0 Antepartum hemorrhage with coagulation defect: O46.0 Intrapartum hemorrhage with coagulation defect: O67.0 Rupture of uterus before onset of labor: O71.0 Rupture of uterus during labor: O71.1 Obstetric shock: O75.1, R57, T80.5, T88.6 Septicemia during labor: O75.3 Puerperal sepsis: O85 Obstetric embolism: O88 Cardiomyopathy in the puerperium: O90.3 , I42, I43 Acute renal failure: O90.4, N17, N19, N99.0 Death, obstetric, cause unspecified: O95 Death, obstetric, after 42 days but 1 year after delivery: O96 Death from sequelae of direct obstetric causes: O97 Sudden death, death from unspecified cause: R96-R99 HIV disease: B20-B24, Z21 Cardiac arrest, cardiac failure, myocardial infarction, or pulmonary edema: O89.1, O74.2, O75.4, I21-I22, I46, I50, J81 Cerebrovascular diseases: subarachnoid and intracranial hemorrhage, cerebral infarction, stroke: I60-I64 Adult respiratory distress syndrome: J80 Acute abdomen: K35, K37, K65, N73.3, N73.5 Hepatic failure: K71-K72 Acute psychosis: F53.1, F23 Cerebral edema or coma: G93.6, R40.2 Disseminated intravascular coagulation: D65 Sickle cell anemia with crisis: D57.0 Status asthmaticus: J45.01, J45.11, J45.81,J45.91 Status epilepticus: G41 |
| Delivery type | Cesarean section: O82 |
| History of substance use disorders | F10-F19 |
| History of neuropsychiatric disorders | F00-F99 |
| **Child characteristics** | |
| Preterm birth | P07.2-P07.3 |
| Low birth weight | P07.0-P07.1 |

*Abbreviation:* ICD-10, international classification of diseases 10th revision.

^*^ Reference: Joseph KS, Liu S, Rouleau J, Kirby RS, Kramer MS, Sauve R, Fraser WD, Young DC, Liston RM. Severe maternal morbidity in Canada, 2003 to 2007: surveillance using routine hospitalization data and ICD-10CA codes. J Obstet Gynaecol Can. 2010 Sep;32(9):837-846. doi: 10.1016/S1701-2163(16)34655-2. PMID: 21050516.

**Table B.** Baseline characteristics of participants in the full unmatched cohort and the sibling comparison cohort,2010-2017.

|  | **Full unmatched cohort** | | **Sibling comparison cohort** | |
| --- | --- | --- | --- | --- |
|  | **(n=2,928,186)** | | **(n=122,948)** | |
|  | **Child with prenatal opioid exposure** | **Children without prenatal exposure** | **Child with prenatal opioid exposure** | **Children without prenatal exposure** |
|  | **(n=118,671)** | **(n=2,809,515)** | **(n=58,850)** | **(n=64,098)** |
| **Maternal characteristics** |  |  |  |  |
| Maternal age at delivery year, mean (SD) | 32 (4.2) | 32 (4.2) | 32 (4.0) | 31 (3.9) |
| Maternal age at delivery year, n (%) |  |  |  |  |
| ≤19 | 344 (0.3) | 7,172 (0.3) | 97 (0.2) | 190 (0.3) |
| 20-24 | 4,469 (3.8) | 123,757 (4.4) | 2,151 (3.7) | 3,853 (6.0) |
| 25-29 | 22,219 (18.7) | 614,597 (21.9) | 11,330 (19.3) | 19,440 (30.3) |
| 30-34 | 56,591 (47.7) | 1,359,683 (48.4) | 28,993 (49.3) | 30,966 (48.3) |
| ≥35 | 35,048 (29.5) | 704,306 (25.1) | 16,279 (27.7) | 9,649 (15.1) |
| Region of residence, n (%) |  |  |  |  |
| Urban | 51,657 (43.5) | 1,247,288 (44.4) | 24,750 (42.1) | 27,747 (43.3) |
| Rural | 67,014 (56.5) | 1,562,227 (55.6) | 34,100 (57.9) | 36,351 (56.7) |
| Income level, n (%) |  |  |  |  |
| 1st quartile | 30,774 (25.9) | 689,080 (24.5) | 13,849 (23.5) | 18,434 (28.8) |
| 2nd quartile | 28,046 (23.6) | 668,723 (23.8) | 13,698 (23.3) | 16,653 (26.0) |
| 3rd quartile | 35,007 (29.5) | 834,862 (29.7) | 18,184 (30.9) | 17,767 (27.7) |
| 4th quartile | 24,844 (20.9) | 616,850 (22.0) | 13,119 (22.3) | 11,244 (17.5) |
| Parity, n (%) |  |  |  |  |
| 1 | 53,144 (44.8) | 1,858,305 (66.1) | 11,136 (18.9) | 43,654 (68.1) |
| ≥2 | 65,527 (55.2) | 951,210 (33.9) | 47,714 (81.1) | 20,444 (31.9) |
| SMM, n (%) |  |  |  |  |
| 0 | 106,762 (90.0) | 2,599,142 (92.5) | 53,107 (90.2) | 57,843 (90.2) |
| 1 | 11,351 (9.6) | 202,942 (7.2) | 5,514 (9.4) | 6,007 (9.4) |
| ≥2 | 558 (0.5) | 7,431 (0.3) | 229 (0.4) | 248 (0.4) |
| Delivery type, n (%) |  |  |  |  |
| Vaginal delivery | 63,205 (53.3) | 1,648,672 (58.7) | 33,289 (56.6) | 38,521 (60.1) |
| Cesarean section | 55,466 (46.7) | 1,160,843 (41.3) | 25,561 (43.4) | 25,577 (39.9) |
| Number of hospital admissions in a year before pregnancy, n (%) |  |  |  |  |
| 0 | 92,676 (78.1) | 2,360,192 (84.0) | 42,581 (72.4) | 51,657 (80.6) |
| 1 | 20,019 (16.9) | 361,444 (12.9) | 12,901 (21.9) | 9,738 (15.2) |
| ≥2 | 5,976 (5.0) | 87,879 (3.1) | 3,368 (5.7) | 2,703 (4.2) |
| Number of outpatient contacts in a year before pregnancy, n (%) |  |  |  |  |
| 0 | 2,694 (2.3) | 189,931 (6.8) | 902 (1.5) | 2,685 (4.2) |
| 1 | 2,629 (2.2) | 155,383 (5.5) | 1,098 (1.9) | 2,276 (3.6) |
| ≥2 | 113,348 (95.5) | 2,464,201 (87.7) | 56,850 (96.6) | 59,137 (92.3) |
| History of neuropsychiatric disorder, n (%) |  |  |  |  |
| No | 103,344 (87.1) | 2,597,509 (92.5) | 51,518 (87.5) | 57,475 (89.7) |
| Yes | 15,327 (12.9) | 212,006 (7.5) | 7,332 (12.5) | 6,623 (10.3) |
| History of substance use disorder, n (%) |  |  |  |  |
| No | 118,357 (99.7) | 2,805,783 (99.9) | 58,718 (99.8) | 63,977 (99.8) |
| Yes | 314 (0.3) | 3,732 (0.1) | 132 (0.2) | 121 (0.2) |
| **Infant characteristics** |  |  |  |  |
| Infant sex, n (%) |  |  |  |  |
| Male | 60,858 (51.3) | 1,442,683 (51.4) | 29,942 (50.9) | 32,565 (50.8) |
| Female | 57,813 (48.7) | 1,366,832 (48.7) | 28,908 (49.1) | 31,533 (49.2) |
| Birth season, n (%) |  |  |  |  |
| Spring | 27,619 (23.3) | 728,982 (26.0) | 13,817 (23.5) | 16,577 (26.0) |
| Summer | 32,424 (27.3) | 685,296 (24.4) | 16,063 (27.3) | 15,619 (24.4) |
| Autumn | 29,461 (24.8) | 694,234 (24.7) | 14,652 (24.9) | 15,808 (24.7) |
| Winter | 29,167 (24.6) | 701,003 (25.0) | 14,318 (24.3) | 16,094 (25.1) |
| Year of delivery, n (%) |  |  |  |  |
| 2010 to 2012 | 39,404 (33.2) | 1,163,452 (41.4) | 12,716 (21.6) | 29,289 (45.7) |
| 2013 to 2015 | 42,194 (35.6) | 1,053,559 (37.5) | 24,543 (41.7) | 27,935 (43.6) |
| 2016 to 2017 | 37,073 (31.2) | 592,504 (21.1) | 21,591 (36.7) | 6,874 (10.7) |
| At-risk newborn, n (%) |  |  |  |  |
| Preterm birth | 6,169 (5.2) | 101,042 (3.6) | 2,540 (4.3) | 1,804 (2.8) |
| Low birth weight | 4,505 (3.8) | 81,352 (2.9) | 1,763 (3.0) | 1,352 (2.1) |

*Abbreviation:* SD, standard deviation; SMM, severe maternal morbidity.

**Table C.** IRR estimates of the association between prenatal opioid exposure and hospital admissions among children using a full unmatched cohort (2010-2017).

|  | **Incidence rate (per 1,000 person-years)^*^** | **Rate difference (per 1,000 person-years)^*^** | **IRR of admission events (95% CI)** | | |
| --- | --- | --- | --- | --- | --- |
|  |  |  | **Crude**^†^ | **Model 1^a^** | **Model 2^b^** |
| Opioid exposure during pregnancy |  |  |  |  |  |
| No | 169.3 | 1 (reference) | 1 (reference) | 1 (reference) | 1 (reference) |
| Yes | 242.5 | 73.2 | **1.43 (1.43, 1.44)** | **1.33 (1.33, 1.34)** | **1.31 (1.30, 1.31)** |
| Timing of opioid exposure |  |  |  |  |  |
| No opioid exposure | 169.3 | 1 (reference) | 1 (reference) | 1 (reference) | 1 (reference) |
| First trimester only | 230.8 | 61.5 | **1.36 (1.36, 1.37)** | **1.27 (1.26, 1.28)** | **1.25 (1.24, 1.26)** |
| Second trimester only | 257.9 | 88.6 | **1.52 (1.51, 1.54)** | **1.38 (1.37, 1.39)** | **1.36 (1.35, 1.37)** |
| Third trimester only | 236.4 | 67.1 | **1.40 (1.39, 1.41)** | **1.29 (1.28, 1.30)** | **1.28 (1.27, 1.29)** |
| More than one trimester | 315.2 | 145.9 | **1.86 (1.84, 1.89)** | **1.33 (1.31, 1.35)** | **1.27 (1.26, 1.29)** |
| Dose-dependent association, MME ^c^ |  |  |  |  |  |
| No opioid exposure | 169.3 | 1 (reference) | 1 (reference) | 1 (reference) | 1 (reference) |
| Low-dose exposure | 221.1 | 51.8 | **1.31 (1.30, 1.31)** | **1.25 (1.24, 1.25)** | **1.23 (1.22, 1.24)** |
| High-dose exposure | 270.9 | 101.6 | **1.60 (1.59, 1.61)** | **1.44 (1.44, 1.45)** | **1.41 (1.40, 1.42)** |
| Days of opioid prescriptions |  |  |  |  |  |
| 0 | 169.3 | 1 (reference) | 1 (reference) | 1 (reference) | 1 (reference) |
| 1-7 | 225.6 | 56.3 | **1.33 (1.33, 1.34)** | **1.26 (1.26, 1.27)** | **1.25 (1.24, 1.25)** |
| 8-11 | 245.3 | 76.0 | **1.45 (1.44, 1.46)** | **1.31 (1.30, 1.33)** | **1.30 (1.29, 1.31)** |
| ≥12 | 282.9 | 113.6 | **1.67 (1.66, 1.68)** | **1.38 (1.37, 1.39)** | **1.34 (1.33, 1.35)** |
| Number of opioid prescriptions |  |  |  |  |  |
| 0-1 | 169.3 | 1 (reference) | 1 (reference) | 1 (reference) | 1 (reference) |
| 2 | 225.0 | 55.7 | **1.33 (1.32, 1.34)** | **1.25 (1.24, 1.26)** | **1.23 (1.23, 1.24)** |
| ≥3 | 263.9 | 94.6 | **1.56 (1.55, 1.57)** | **1.36 (1.35, 1.37)** | **1.33 (1.33, 1.34)** |
| Maternal health condition with opioid prescriptions |  |  |  |  |  |
| No opioid exposure | 169.3 | 1 (reference) | 1 (reference) | 1 (reference) | 1 (reference) |
| Pain-related ^d^ | 270.6 | 101.3 | **1.60 (1.58, 1.62)** | **1.37 (1.36, 1.39)** | **1.33 (1.31, 1.34)** |
| Antitussive-related ^e^ | 237.1 | 67.8 | **1.40 (1.39, 1.41)** | **1.30 (1.30, 1.31)** | **1.29 (1.28, 1.29)** |
| Other | 247.0 | 77.7 | **1.46 (1.45, 1.47)** | **1.27 (1.26, 1.28)** | **1.25 (1.24, 1.26)** |
| Opioid type |  |  |  |  |  |
| No opioid exposure | 169.3 | 1 (reference) | 1 (reference) | 1 (reference) | 1 (reference) |
| Codeine | 257.8 | 88.5 | **1.52 (1.13, 2.05)** | 1.17 (0.87, 1.57) | 1.08 (0.80, 1.45) |
| Dihydrocodeine | 233.5 | 64.2 | **1.38 (1.37, 1.39)** | **1.30 (1.29, 1.30)** | **1.28 (1.27, 1.28)** |
| Fentanyl | 204.3 | 35.0 | **1.21 (1.17, 1.25)** | **1.12 (1.09, 1.16)** | **1.10 (1.07, 1.14)** |
| Morphine | 297.4 | 128.1 | **1.76 (1.70, 1.82)** | **1.24 (1.20, 1.28)** | **1.23 (1.19, 1.27)** |
| Oxycodone | 221.0 | 51.7 | **1.31 (1.24, 1.37)** | **1.28 (1.22, 1.34)** | **1.27 (1.21, 1.34)** |
| Tramadol | 215.7 | 46.4 | **1.27 (1.26, 1.29)** | **1.23 (1.22, 1.25)** | **1.21 (1.20, 1.23)** |
| Polytherapy | 286.6 | 117.3 | **1.69 (1.68, 1.71)** | **1.37 (1.36, 1.38)** | **1.33 (1.32, 1.34)** |

*Abbreviation:* IRR, incidence rate ratio; CI, confidence interval; MME, morphine milligram equivalent; SMM, severe maternal morbidity.

Numbers in bold indicate significant differences (*p*<0.05).

^*^ Incidence rates were calculated as the number of hospital admissions per 1,000 person-years. Rate differences represent the absolute difference in incidence rates between the opioid-exposed and unexposed groups per 1,000 person-years.

^†^ Crude estimates were unadjusted.

**^a^ Model 1** **was adjusted for maternal age at delivery; infant sex; region of residence (urban and rural); household income level (1st, 2nd, 3rd, and 4th quartiles); birth season (spring, summer, autumn, and winter); parity (1 and ≥2); SMM score;** number of hospital outpatient visits and admissions in the year before pregnancy**; preterm birth; low birth weight; delivery type (vaginal delivery or cesarean section); and year of delivery (2010-2012, 2013-2015, and 2016-2017).**

**^b^** **Model 2 was adjusted for all variables in Model 1,** with further adjustment for maternal history of substance use disorders and neuropsychiatric disorders, and plurality (number of infants per delivery).

^c^ Low and high doses were divided based on the median value (18 MME).

^d^ Pain-related condition includes medical conditions such as neoplasm (benign), diseases of the blood and blood-forming organs and certain disorders involving the immune mechanism, endocrine, nutritional and metabolic diseases, neuropsychiatric disorders, diseases of the nervous system, diseases of the ear and mastoid process, diseases of the circulatory system, diseases of digestive system, diseases of the skin and subcutaneous tissue, diseases of musculoskeletal system and connective tissue, and diseases of the genitourinary system.

^e^ Antitussive-related conditions include medical conditions such as infectious and parasitic diseases, and diseases of the respiratory system.

**Table D.** IRR estimates of the dose-dependent association between prenatal opioid exposure and hospital admissions among children, using a full unmatched cohort (2010-2017).

|  | **Overall** | | | | **Low-dose exposure (≤18 MME)** | | | | **High-dose exposure (>18 MME)** | | | | |
| --- | --- | --- | --- | --- | --- | --- | --- | --- | --- | --- | --- | --- | --- |
|  | Incidence rate (per 1,000 PY)^*^ | **Crude^†^** | **Model 1^a^** | **Model 2^b^** | Incidence rate (per 1,000 PY)^*^ | **Crude^†^** | **Model 1^a^** | **Model 2^b^** | Incidence rate (per 1,000 PY)^*^ | **Crude^†^** | **Model 1^a^** | **Model 2^b^** |  |
| Hospital admission |  |  |  |  |  |  |  |  |  |  |  |  |  |
| No opioid exposure | 169.3 | 1 (reference) | 1 (reference) | 1 (reference) | 169.3 | 1 (reference) | 1 (reference) | 1 (reference) | 169.3 | 1 (reference) | 1 (reference) | 1 (reference) |  |
| Opioid exposure | 242.5 | **1.43 (1.43, 1.44)** | **1.33 (1.33, 1.34)** | **1.31 (1.30, 1.31)** | 221.1 | **1.31 (1.30, 1.31)** | **1.25 (1.24, 1.26)** | **1.23 (1.22, 1.24)** | 270.9 | **1.60 (1.59, 1.61)** | **1.44 (1.44, 1.45)** | **1.41 (1.40, 1.42)** |  |
| Infection |  |  |  |  |  |  |  |  |  |  |  |  |  |
| No opioid exposure | 147.9 | 1 (reference) | 1 (reference) | 1 (reference) | 147.9 | 1 (reference) | 1 (reference) | 1 (reference) | 147.9 | 1 (reference) | 1 (reference) | 1 (reference) |  |
| Opioid exposure | 215.7 | **1.46 (1.45, 1.46)** | **1.36 (1.35, 1.36)** | **1.33 (1.32, 1.33)** | 195.5 | **1.32 (1.31, 1.33)** | **1.26 (1.26, 1.27)** | **1.24 (1.24, 1.25)** | 242.5 | **1.64 (1.63, 1.65)** | **1.48 (1.47, 1.48)** | **1.44 (1.43, 1.44)** |  |
| Respiratory non-infection |  |  |  |  |  |  |  |  |  |  |  |  |  |
| No opioid exposure | 5.1 | 1 (reference) | 1 (reference) | 1 (reference) | 5.1 | 1 (reference) | 1 (reference) | 1 (reference) | 5.1 | 1 (reference) | 1 (reference) | 1 (reference) |  |
| Opioid exposure | 7.3 | **1.44 (1.41, 1.47)** | **1.36 (1.33, 1.39)** | **1.33 (1.30, 1.36)** | 6.5 | **1.28 (1.24, 1.32)** | **1.24 (1.20, 1.28)** | **1.22 (1.18, 1.26)** | 8.3 | **1.64 (1.59, 1.69)** | **1.52 (1.47, 1.56)** | **1.48 (1.43, 1.52)** |  |
| Gastrointestinal non-infection |  |  |  |  |  |  |  |  |  |  |  |  |  |
| No opioid exposure | 4.4 | 1 (reference) | 1 (reference) | 1 (reference) | 4.4 | 1 (reference) | 1 (reference) | 1 (reference) | 4.4 | 1 (reference) | 1 (reference) | 1 (reference) |  |
| Opioid exposure | 5.3 | **1.19 (1.16, 1.22)** | **1.15 (1.12, 1.18)** | **1.13 (1.10, 1.16)** | 5.1 | **1.16 (1.12, 1.20)** | **1.13 (1.09, 1.17)** | **1.12 (1.08, 1.16)** | 5.5 | **1.24 (1.19, 1.29)** | **1.17 (1.12, 1.21)** | **1.15 (1.10, 1.19)** |  |
| Oral cavity |  |  |  |  |  |  |  |  |  |  |  |  |  |
| No opioid exposure | 0.6 | 1 (reference) | 1 (reference) | 1 (reference) | 0.6 | 1 (reference) | 1 (reference) | 1 (reference) | 0.6 | 1 (reference) | 1 (reference) | 1 (reference) |  |
| Opioid exposure | 0.9 | **1.40 (1.32, 1.50)** | **1.32 (1.24, 1.41)** | **1.29 (1.21, 1.38)** | 0.9 | **1.37 (1.26, 1.50)** | **1.33 (1.22, 1.45)** | **1.31 (1.20, 1.42)** | 0.9 | **1.44 (1.31, 1.59)** | **1.31 (1.19, 1.45)** | **1.28 (1.16, 1.41)** |  |
| Injury/external |  |  |  |  |  |  |  |  |  |  |  |  |  |
| No opioid exposure | 10.4 | 1 (reference) | 1 (reference) | 1 (reference) | 10.4 | 1 (reference) | 1 (reference) | 1 (reference) | 10.4 | 1 (reference) | 1 (reference) | 1 (reference) |  |
| Opioid exposure | 12.3 | **1.19 (1.17, 1.21)** | **1.13 (1.11, 1.15)** | **1.11 (1.10, 1.13)** | 12.1 | **1.17 (1.14, 1.19)** | **1.12 (1.10, 1.15)** | **1.11 (1.09, 1.14)** | 12.7 | **1.22 (1.19, 1.25)** | **1.13 (1.11, 1.16)** | **1.12 (1.09, 1.15)** |  |
| Genitourinary non-infection |  |  |  |  |  |  |  |  |  |  |  |  |  |
| No opioid exposure | 5.1 | 1 (reference) | 1 (reference) | 1 (reference) | 5.1 | 1 (reference) | 1 (reference) | 1 (reference) | 5.1 | 1 (reference) | 1 (reference) | 1 (reference) |  |
| Opioid exposure | 6.2 | **1.22 (1.19, 1.25)** | **1.16 (1.14, 1.19)** | **1.15 (1.12, 1.18)** | 5.8 | **1.14 (1.10, 1.17)** | **1.11 (1.07, 1.14)** | **1.10 (1.06, 1.13)** | 6.8 | **1.32 (1.28, 1.37)** | **1.24 (1.20, 1.28)** | **1.22 (1.18, 1.26)** |  |

*Abbreviation:* IRR, incidence rate ratio; CI, confidence interval; MME, morphine milligram equivalent; PY, person-year; SMM, severe maternal morbidity.

Numbers in bold indicate significant differences (*p*<0.05).

Low and high dose were divided based on median value (18 MME).

^*^ Incidence rates were calculated as the number of hospital admissions per 1,000 person-years.

^†^ Crude estimates were unadjusted.

^a^ Model 1 was adjusted for maternal age at delivery; infant sex; region of residence (urban and rural); household income level (1st, 2nd, 3rd, and 4th quartiles); birth season (spring, summer, autumn, and winter); parity (1 and ≥2); SMM score; number of hospital outpatient visits and admissions in the year before pregnancy; preterm birth; low birth weight; delivery type (vaginal delivery or cesarean section); and year of delivery (2010-2012, 2013-2015, and 2016-2017).

**^b^ Model 2 was adjusted for all variables in Model 1,** with further adjustment for maternal history of substance use disorders and neuropsychiatric disorders, and plurality (number of infants per delivery).

**Table E.** IRR estimates of the dose-dependent association between prenatal opioid exposure and hospital admissions among children, using a sibling comparison cohort (2010-2017).

|  | **Overall** | | | | **Low-dose exposure (≤18 MME)** | | | | **High-dose exposure (>18 MME)** | | | |
| --- | --- | --- | --- | --- | --- | --- | --- | --- | --- | --- | --- | --- |
|  | **Incidence rate (per 1,000 PY)^*^** | **Crude^†^** | **Model 1^a^** | **Model 2^b^** | **Incidence rate (per 1,000 PY)^*^** | **Crude^†^** | **Model 1^a^** | **Model 2^b^** | **Incidence rate (per 1,000 PY)^*^** | **Crude^†^** | **Model 1^a^** | **Model 2^b^** |
| Hospital admission |  |  |  |  |  |  |  |  |  |  |  |  |
| No opioid exposure | 216.9 | 1 (reference) | 1 (reference) | 1 (reference) | 216.9 | 1 (reference) | 1 (reference) | 1 (reference) | 216.9 | 1 (reference) | 1 (reference) | 1 (reference) |
| Opioid exposure | 233.9 | **1.20 (1.17, 1.22)** | **1.09 (1.07, 1.10)** | **1.08 (1.07, 1.10)** | 212.7 | **1.08 (1.04, 1.11)** | 1.00 (0.98, 1.01) | 1.00 (0.98, 1.01) | 259.7 | **1.32 (1.28, 1.35)** | **1.19 (1.18, 1.21)** | **1.19 (1.17, 1.20)** |
| Infection |  |  |  |  |  |  |  |  |  |  |  |  |
| No opioid exposure | 203.8 | 1 (reference) | 1 (reference) | 1 (reference) | 203.8 | 1 (reference) | 1 (reference) | 1 (reference) | 203.8 | 1 (reference) | 1 (reference) | 1 (reference) |
| Opioid exposure | 219.9 | **1.20 (1.17, 1.23)** | **1.09 (1.07, 1.10)** | **1.07 (1.05, 1.08)** | 199.7 | **1.07 (1.04, 1.11)** | 0.99 (0.98, 1.01) | 0.99 (0.97, 1.00) | 244.4 | **1.33 (1.29, 1.37)** | **1.20 (1.18, 1.22)** | **1.17 (1.15, 1.19)** |
| Respiratory non-infection |  |  |  |  |  |  |  |  |  |  |  |  |
| No opioid exposure | 7.3 | 1 (reference) | 1 (reference) | 1 (reference) | 7.3 | 1 (reference) | 1 (reference) | 1 (reference) | 7.3 | 1 (reference) | 1 (reference) | 1 (reference) |
| Opioid exposure | 6.9 | 1.00 (0.88, 1.14) | 0.99 (0.91, 1.08) | 0.98 (0.90, 1.06) | 6.2 | 0.89 (0.73, 1.07) | **0.90 (0.81, 0.99)** | **0.89 (0.81, 0.98)** | 7.7 | 1.11 (0.94, 1.32) | **1.11 (1.01, 1.22)** | 1.08 (0.98, 1.19) |
| Gastrointestinal non-infection |  |  |  |  |  |  |  |  |  |  |  |  |
| No opioid exposure | 4.8 | 1 (reference) | 1 (reference) | 1 (reference) | 4.8 | 1 (reference) | 1 (reference) | 1 (reference) | 4.8 | 1 (reference) | 1 (reference) | 1 (reference) |
| Opioid exposure | 5.0 | 1.11 (0.96, 1.29) | 1.08 (0.98, 1.20) | 1.07 (0.96, 1.18) | 4.7 | 1.06 (0.85, 1.31) | 1.04 (0.93, 1.16) | 1.03 (0.92, 1.15) | 5.3 | 1.17 (0.96, 1.44) | **1.14 (1.02, 1.28)** | 1.11 (0.99, 1.25) |
| Oral cavity |  |  |  |  |  |  |  |  |  |  |  |  |
| No opioid exposure | 0.8 | 1 (reference) | 1 (reference) | 1 (reference) | 0.8 | 1 (reference) | 1 (reference) | 1 (reference) | 0.8 | 1 (reference) | 1 (reference) | 1 (reference) |
| Opioid exposure | 0.9 | 1.22 (0.86, 1.73) | 1.19 (0.93, 1.51) | 1.17 (0.91, 1.49) | 0.9 | 1.15 (0.69, 1.90) | 1.12 (0.86, 1.47) | 1.12 (0.86, 1.47) | 1.0 | 1.28 (0.80, 2.06) | 1.27 (0.96, 1.67) | 1.22 (0.93, 1.61) |
| Injury/external |  |  |  |  |  |  |  |  |  |  |  |  |
| No opioid exposure | 11.7 | 1 (reference) | 1 (reference) | 1 (reference) | 11.7 | 1 (reference) | 1 (reference) | 1 (reference) | 11.7 | 1 (reference) | 1 (reference) | 1 (reference) |
| Opioid exposure | 13.1 | **1.18 (1.08, 1.30)** | **1.08 (1.01, 1.15)** | **1.07 (1.01, 1.14)** | 13.0 | **1.17 (1.02, 1.34)** | 1.07 (1.00, 1.15) | 1.07 (1.00, 1.15) | 13.2 | **1.20 (1.05, 1.37)** | **1.09 (1.01, 1.17)** | **1.08 (1.00, 1.16)** |
| Genitourinary non-infection |  |  |  |  |  |  |  |  |  |  |  |  |
| No opioid exposure | 5.4 | 1 (reference) | 1 (reference) | 1 (reference) | 5.4 | 1 (reference) | 1 (reference) | 1 (reference) | 5.4 | 1 (reference) | 1 (reference) | 1 (reference) |
| Opioid exposure | 6.5 | **1.36 (1.19, 1.54)** | **1.18 (1.07, 1.29)** | **1.17 (1.07, 1.28)** | 6.0 | **1.23 (1.01, 1.48)** | 1.09 (0.98, 1.20) | 1.09 (0.98, 1.20) | 7.2 | **1.48 (1.25, 1.76)** | **1.29 (1.16, 1.43)** | **1.27 (1.15, 1.41)** |

*Abbreviation:* IRR, incidence rate ratio; CI, confidence interval; MME, morphine milligram equivalent; PY, person-year; SMM, severe maternal morbidity.

Numbers in bold indicate significant differences (*p*<0.05).

Low and high dose were divided based on median value (18 MME).

^*^ Incidence rates were calculated as the number of hospital admissions per 1,000 person-years.

^†^ Crude estimates were obtained from conditional Poisson models stratified on family, including prenatal opioid exposure and the person-year offset only, without further covariate adjustment.

^a^ Model 1 was adjusted for maternal age at delivery; infant sex; region of residence (urban and rural); household income level (1st, 2nd, 3rd, and 4th quartiles); birth season (spring, summer, autumn, and winter); parity (1 and ≥2); SMM score; number of hospital outpatient visits and admissions in the year before pregnancy; preterm birth; low birth weight; delivery type (vaginal delivery or cesarean section); and year of delivery (2010-2012, 2013-2015, and 2016-2017).

**^b^ Model 2 was adjusted for all variables in Model 1,** with further adjustment for maternal history of substance use disorders and neuropsychiatric disorders, and plurality (number of infants per delivery).

**Table F.** Baseline characteristics of participants in the PS-matched sibling comparison cohort, 2010-2017.

|  | **PS-matched sibling comparison cohort ^a^** | | |
| --- | --- | --- | --- |
|  | **(n=27,327)** | | |
|  | **Child without prenatal opioid exposure** | **Children with prenatal exposure** | **SMD^*^** |
|  | **(n=** **13,739)** | **(n=13,588)** |  |
| **Maternal characteristics** |  |  |  |
| Maternal age at delivery year, mean (SD) | 31 (4.0) | 32 (4.1) | 0.08 |
| Maternal age at delivery year, n (%) |  |  | 0.06 |
| ≤19 | 44 (0.3) | 34 (0.3) |  |
| 20-24 | 641 (4.7) | 623 (4.6) |  |
| 25-29 | 3,466 (25.2) | 3,068 (22.6) |  |
| 30-34 | 6,605 (48.1) | 6,466 (47.6) |  |
| ≥35 | 2,983 (21.7) | 3,397 (25.0) |  |
| Region of residence, n (%) |  |  | <0.01 |
| Urban | 5,748 (41.8) | 5,625 (41.4) |  |
| Rural | 7,991 (58.2) | 7,963 (58.6) |  |
| Income level, n (%) |  |  | 0.07 |
| 1st quartile | 4,127 (30.0) | 3,775 (27.8) |  |
| 2nd quartile | 3,457 (25.2) | 3,295 (24.3) |  |
| 3rd quartile | 3,757 (27.4) | 3,865 (28.4) |  |
| 4th quartile | 2,398 (17.5) | 2,653 (19.5) |  |
| Parity, n (%) |  |  | 0.20 |
| 1 | 5,924 (43.1) | 4,525 (33.3) |  |
| ≥2 | 7,815 (56.9) | 9,063 (66.7) |  |
| SMM, n (%) |  |  | <0.01 |
| 0 | 12,129 (88.3) | 12,013 (88.4) |  |
| 1 | 1,527 (11.1) | 1,493 (11.0) |  |
| ≥2 | 83 (0.6) | 82 (0.6) |  |
| Delivery type, n (%) |  |  | 0.02 |
| Vaginal delivery | 7,839 (57.1) | 7,615 (56.0) |  |
| Cesarean section | 5,900 (42.9) | 5,973 (44.0) |  |
| Number of hospital admissions in a year before pregnancy, n (%) |  |  | 0.03 |
| 0 | 10,159 (73.9) | 9,867 (72.6) |  |
| 1 | 2,703 (19.7) | 2,919 (21.5) |  |
| ≥2 | 877 (6.4) | 802 (5.9) |  |
| Number of outpatient contacts in a year before pregnancy, n (%) |  |  | 0.12 |
| 0 | 153 (1.1) | 239 (1.8) |  |
| 1 | 170 (1.2) | 296 (2.2) |  |
| ≥2 | 13,416 (97.7) | 13,053 (96.1) |  |
| History of neuropsychiatric disorder, n (%) |  |  | <0.01 |
| No | 11,510 (83.8) | 11,402 (83.9) |  |
| Yes | 2,229 (16.2) | 2,186 (16.1) |  |
| History of substance use disorder, n (%) |  |  | <0.01 |
| No | 13,702 (99.7) | 13,548 (99.7) |  |
| Yes | 37 (0.3) | 40 (0.3) |  |
| **Infant characteristics** |  |  |  |
| Infant sex, n (%) |  |  | <0.01 |
| Male | 7,065 (51.4) | 6,846 (50.4) |  |
| Female | 6,674 (48.6) | 6,742 (49.6) |  |
| Birth season, n (%) |  |  | 0.03 |
| Spring | 3,173 (23.1) | 3,141 (23.1) |  |
| Summer | 3,770 (27.4) | 3,732 (27.5) |  |
| Autumn | 3,369 (24.5) | 3,329 (24.5) |  |
| Winter | 3,427 (24.9) | 3,386 (24.9) |  |
| Year of delivery, n (%) |  |  | 0.15 |
| 2010 to 2012 | 4,743 (34.5) | 3,961 (29.2) |  |
| 2013 to 2015 | 5,722 (41.7) | 5,777 (42.5) |  |
| 2016 to 2017 | 3,274 (23.8) | 3,850 (28.3) |  |
| At-risk newborn, n (%) |  |  |  |
| Preterm birth | 503 (3.7) | 592 (4.4) | 0.04 |
| Low birth weight | 319 (2.3) | 409 (3.0) | 0.04 |

*Abbreviation:* PS, propensity score; SD, standard deviation; SMD, standardized mean difference; SMM, severe maternal morbidity.

^*^ SMD <0.1 corresponds to no major imbalance.

^a^ PS-matched sibling comparison cohort, derived from the 1:5 PS-matched cohort.

**Table G.** IRR estimates of the association between prenatal opioid exposure and hospital admissions among children using a PS-matched sibling comparison cohort (2010-2017).

|  | **Incidence rate (per 1,000 person-years)^*^** | **Rate difference (per 1,000 person-years)^*^** | **IRR of admission events (95% CI)** | | |
| --- | --- | --- | --- | --- | --- |
|  |  |  | **Crude**^†^ | **Model 1^a^** | **Model 2^b^** |
| Opioid exposure during pregnancy |  |  |  |  |  |
| No | 244.3 | 1 (reference) | 1 (reference) | 1 (reference) | 1 (reference) |
| Yes | 247.8 | 3.5 | **1.13 (1.08, 1.18)** | **1.07 (1.02, 1.12)** | **1.07 (1.02, 1.12)** |
| Timing of opioid exposure |  |  |  |  |  |
| No opioid exposure | 244.3 | 1 (reference) | 1 (reference) | 1 (reference) | 1 (reference) |
| First trimester only | 234.8 | -9.5 | 1.05 (0.98, 1.13) | 1.00 (0.93, 1.07) | 1.00 (0.93, 1.07) |
| Second trimester only | 260.9 | 16.6 | **1.19 (1.08, 1.31)** | **1.12 (1.02, 1.23)** | **1.12 (1.02, 1.23)** |
| Third trimester only | 242.0 | -2.3 | **1.09 (1.00, 1.19)** | 1.04 (0.95, 1.14) | 1.04 (0.95, 1.14) |
| More than one trimester | 345.8 | 101.5 | **1.64 (1.42, 1.89)** | **1.52 (1.32, 1.76)** | **1.53 (1.32, 1.77)** |
| Dose-dependent association, MME ^c^ |  |  |  |  |  |
| No opioid exposure | 244.3 | 1 (reference) | 1 (reference) | 1 (reference) | 1 (reference) |
| Low-dose exposure | 227.6 | -16.7 | 1.01 (0.95, 1.08) | 0.97 (0.91, 1.03) | 0.97 (0.91, 1.04) |
| High-dose exposure | 275.7 | 31.4 | **1.26 (1.19, 1.34)** | **1.18 (1.11, 1.25)** | **1.18 (1.11, 1.26)** |
| Days of opioid prescriptions |  |  |  |  |  |
| 0 | 244.3 | 1 (reference) | 1 (reference) | 1 (reference) | 1 (reference) |
| 1-7 | 230.2 | -14.1 | 1.02 (0.96, 1.09) | 0.98 (0.92, 1.04) | 0.98 (0.92, 1.04) |
| 8-11 | 257.8 | 13.5 | **1.14 (1.04, 1.25)** | 1.08 (0.98, 1.19) | 1.08 (0.98, 1.19) |
| ≥12 | 285.1 | 40.8 | **1.35 (1.24, 1.46)** | **1.25 (1.15, 1.36)** | **1.26 (1.16, 1.36)** |
| Number of opioid prescriptions |  |  |  |  |  |
| 0-1 | 244.3 | 1 (reference) | 1 (reference) | 1 (reference) | 1 (reference) |
| 2 | 228.6 | -15.7 | 1.02 (0.96, 1.09) | 0.98 (0.91, 1.04) | 0.98 (0.92, 1.04) |
| ≥3 | 271.7 | 27.4 | **1.25 (1.17, 1.33)** | **1.17 (1.10, 1.24)** | **1.17 (1.10, 1.24)** |
| Maternal health condition with opioid prescriptions |  |  |  |  |  |
| No opioid exposure | 244.3 | 1 (reference) | 1 (reference) | 1 (reference) | 1 (reference) |
| Pain-related ^d^ | 270.0 | 25.7 | **1.27 (1.10, 1.47)** | **1.18 (1.02, 1.36)** | **1.18 (1.02, 1.36)** |
| Antitussive-related ^e^ | 242.7 | -1.6 | **1.10 (1.04, 1.16)** | 1.04 (0.98, 1.10) | 1.04 (0.98, 1.10) |
| Other | 253.3 | 9.0 | **1.16 (1.06, 1.26)** | **1.10 (1.01, 1.20)** | **1.10 (1.02, 1.20)** |
| Opioid type |  |  |  |  |  |
| No opioid exposure | 244.3 | 1 (reference) | 1 (reference) | 1 (reference) | 1 (reference) |
| Codeine | 80.0 | -164.3 | 0.33 (0.00, 1213.91) | 0.17 (0.00, 635.57) | 0.17 (0.00, 632.51) |
| Dihydrocodeine | 241.7 | -2.6 | **1.09 (1.03, 1.15)** | 1.04 (0.98, 1.10) | 1.04 (0.98, 1.10) |
| Fentanyl | 199.0 | -45.3 | 0.91 (0.59, 1.39) | 0.87 (0.57, 1.33) | 0.86 (0.56, 1.32) |
| Morphine | 229.0 | -15.3 | 1.04 (0.72, 1.49) | 0.97 (0.68, 1.40) | 0.98 (0.68, 1.41) |
| Oxycodone | 264.0 | 19.7 | 1.11 (0.52, 2.35) | 0.99 (0.47, 2.10) | 0.99 (0.47, 2.10) |
| Tramadol | 223.4 | -20.9 | 1.05 (0.90, 1.22) | 0.99 (0.85, 1.15) | 0.99 (0.85, 1.16) |
| Polytherapy | 285.0 | 40.7 | **1.30 (1.20, 1.42)** | **1.21 (1.11, 1.32)** | **1.21 (1.11, 1.32)** |

*Abbreviation:* IRR, incidence rate ratio; CI, confidence interval; PS, propensity score; SMM, severe maternal morbidity.

Numbers in bold indicate significant differences (*p*<0.05).

^*^ Incidence rates were calculated as the number of hospital admissions per 1,000 person-years. Rate differences represent the absolute difference in incidence rates between the opioid-exposed and unexposed groups per 1,000 person-years.

^†^ Crude estimates were obtained from conditional Poisson models stratified on family, including prenatal opioid exposure and the person-year offset only, without further covariate adjustment.

**^a^ Model 1 was adjusted for maternal age at delivery; infant sex; region of residence (urban and rural); household income level (1st, 2nd, 3rd, and 4th quartiles); birth season (spring, summer, autumn, and winter); parity (1 and ≥2); SMM score;** number of hospital outpatient visits and admissions in the year before pregnancy**; preterm birth; low birth weight; delivery type (vaginal delivery or cesarean section); and year of delivery (2010-2012, 2013-2015, and 2016-2017).**

**^b^ Model 2 was adjusted for all variables in Model 1,** with further adjustment for maternal history of substance use disorders and neuropsychiatric disorders, and plurality (number of infants per delivery).

^c^ Low and high doses were divided based on the median value (18 MME).

^d^ Pain-related condition includes medical conditions such as neoplasm (benign), diseases of the blood and blood-forming organs and certain disorders involving the immune mechanism, endocrine, nutritional and metabolic diseases, neuropsychiatric disorders, diseases of the nervous system, diseases of the ear and mastoid process, diseases of the circulatory system, diseases of digestive system, diseases of the skin and subcutaneous tissue, diseases of musculoskeletal system and connective tissue, and diseases of the genitourinary system.

^e^ Antitussive-related conditions include medical conditions such as infectious and parasitic diseases, and diseases of the respiratory system.

**Table H.** Sensitivity analysis excluding potential mediators from the association between prenatal opioid exposure and hospital admissions among children using a 1:5 PS-matched cohort and sibling comparison cohort (2010-2017).

|  | **1:5 PS-matched cohort** | | **Sibling comparison cohort** | |
| --- | --- | --- | --- | --- |
|  | **aIRR, Model 1^a,c^** | **aIRR, Model 2^b,c^** | **aIRR, Model 1^a,c^** | **aIRR, Model 2^b,c^** |
| **Overall** | **1.29 (1.28, 1.29)** | **1.29 (1.28, 1.29)** | **1.08 (1.07, 1.10)** | **1.08 (1.07, 1.10)** |
| **Low-dose exposure (≤18 MME)** | **1.22 (1.21, 1.22)** | **1.22 (1.21, 1.23)** | 0.99 (0.98, 1.01) | 1.00 (0.98, 1.01) |
| **High-dose exposure (>18 MME)** | **1.37 (1.36, 1.38)** | **1.37 (1.36, 1.38)** | **1.19 (1.17, 1.21)** | **1.19 (1.17, 1.21)** |

*Abbreviation:* aIRR, adjusted incidence rate ratio; MME, morphine milligram equivalent; SMM, severe maternal morbidity.

Numbers in bold indicate significant differences (*p*<0.05).

Low and high dose were divided based on median value (18 MME).

^a^ Model 1 was adjusted for maternal age at delivery; infant sex; region of residence (urban and rural); household income level (1st, 2nd, 3rd, and 4th quartiles); birth season (spring, summer, autumn, and winter); parity (1 and ≥2); SMM score; number of hospital outpatient visits and admissions in the year before pregnancy; and year of delivery (2010-2012, 2013-2015, and 2016-2017).

**^b^ Model 2 was adjusted for all variables in Model 1,** with further adjustment for maternal history of substance use disorders and neuropsychiatric disorders, and plurality (number of infants per delivery).

^c^ Preterm birth, low birth weight, and delivery type were excluded from both models to evaluate the potential impact of adjusting for variables that may act as mediators on the causal pathway between prenatal opioid exposure and hospital admissions.

**Table I.** Sensitivity analysis of the association between prenatal opioid exposure and hospital admissions among children after excluding pregnancies with a single opioid prescription from the reference group (2010–2017).

|  | **IRR of admission events (95% CI)** | | | |
| --- | --- | --- | --- | --- |
|  | **Crude**^*^ | **Model 1^a^** | **Model 2^b^** |  |
| **Full unmatched cohort** (n=2,116,030; 812,156 [28.9%] of the reference group excluded) |  |  |  |  |
| No opioid exposure (0 prescriptions) | 1 (reference) | 1 (reference) | 1 (reference) |  |
| Prenatal opioid exposure (≥2 prescriptions) | **1.59 (1.58, 1.60)** | **1.45 (1.44, 1.45)** | **1.41 (1.41, 1.42)** |  |
| Low-dose exposure (≤18 MME)^e^ | **1.45 (1.44, 1.46)** | **1.34 (1.34, 1.35)** | **1.32 (1.31, 1.32)** |  |
| High-dose exposure (>18 MME)^e^ | **1.77 (1.77, 1.78)** | **1.59 (1.58, 1.60)** | **1.54 (1.54, 1.55)** |  |
| **1:5 PS-matched cohort, re-matched** (n=407,184)^c^ |  |  |  |  |
| No opioid exposure (0 prescriptions) | 1 (reference) | 1 (reference) | 1 (reference) |  |
| Prenatal opioid exposure (≥2 prescriptions) | **1.28 (1.28, 1.29)** | **1.25 (1.24, 1.26)** | **1.25 (1.24, 1.26)** |  |
| Low-dose exposure (≤18 MME)^e^ | **1.19 (1.18, 1.20)** | **1.17 (1.16, 1.18)** | **1.17 (1.16, 1.17)** |  |
| High-dose exposure (>18 MME)^e^ | **1.43 (1.42, 1.44)** | **1.39 (1.37, 1.40)** | **1.38 (1.37, 1.39)** |  |
| **Sibling comparison cohort, re-derived** (n=76,536)^d^ |  |  |  |  |
| No opioid exposure (0 prescriptions) | 1 (reference) | 1 (reference) | 1 (reference) |  |
| Prenatal opioid exposure (≥2 prescriptions) | **1.28 (1.26, 1.30)** | **1.05 (1.03, 1.08)** | **1.05 (1.02, 1.07)** |  |
| Low-dose exposure (≤18 MME)^e^ | **1.16 (1.14, 1.18)** | **0.97 (0.94, 0.99)** | **0.97 (0.94, 0.99)** |  |
| High-dose exposure (>18 MME)^e^ | **1.40 (1.38, 1.43)** | **1.15 (1.12, 1.18)** | **1.15 (1.12, 1.17)** |  |

*Abbreviation:* IRR, incidence rate ratio; CI, confidence interval; MME, morphine milligram equivalent; PS, propensity score; SMM, severe maternal morbidity.

Numbers in bold indicate significant differences (*p*<0.05).

Under the primary exposure definition, prenatal opioid exposure was defined as maternal receipt of two or more opioid prescriptions within any given trimester; pregnancies with a single opioid prescription were therefore classified as unexposed. In this sensitivity analysis, these pregnancies were excluded so that the reference group comprised only pregnancies with no opioid prescription.

^*^ Crude estimates were derived from unadjusted models containing prenatal opioid exposure and the person-year offset only; in the sibling comparison cohort, models were additionally stratified on family.

**^a^ Model 1 was adjusted for maternal age at delivery; infant sex; region of residence (urban and rural); household income level (1st, 2nd, 3rd, and 4th quartiles); birth season (spring, summer, autumn, and winter); parity (1 and ≥2); SMM score;** number of hospital outpatient visits and admissions in the year before pregnancy**; preterm birth; low birth weight; delivery type (vaginal delivery or cesarean section); and year of delivery (2010-2012, 2013-2015, and 2016-2017).**

**^b^ Model 2 was adjusted for all variables in Model 1,** with further adjustment for maternal history of substance use disorders and neuropsychiatric disorders, and plurality (number of infants per delivery).

^c^ Propensity score matching was repeated after exclusion of pregnancies with a single opioid prescription. As the matched cohort was reconstructed rather than restricted, the number excluded is not comparable with the other cohorts and is not reported; fewer exposed pregnancies could be matched, reflecting reduced propensity score overlap after the exclusion.

^d^ Discordant sibships were re-identified after exclusion of pregnancies with a single opioid prescription; families in which all remaining siblings shared the same exposure status were no longer eligible.

^e^ Low and high doses were divided based on the median value (18 MME).

**Table J.** Stratified IRR estimates of the association between prenatal opioid exposure and hospital admissions among children using a 1:5 PS-matched cohort (2010-2017).

|  | **Incidence rate (per 1,000 person-years)^*^** | **Rate difference (per 1,000 person-years)^*^** | **IRR of admission events (95% CI)** | | |  |
| --- | --- | --- | --- | --- | --- | --- |
|  |  |  | **Crude**^†^ | **Model 1^a^** | **Model 2^b^** | |
| **Maternal characteristics** |  |  |  |  |  | |
| Maternal age at delivery year, n (%) |  |  |  |  |  | |
| ≤19 | 342.3 | 98.4 | **1.40 (1.31, 1.50)** | **1.28 (1.20, 1.37)** | **1.31 (1.23, 1.41)** | |
| 20-24 | 328.3 | 88.3 | **1.37 (1.34, 1.39)** | **1.32 (1.29, 1.34)** | **1.32 (1.29, 1.34)** | |
| 25-29 | 267.6 | 61.4 | **1.30 (1.29, 1.31)** | **1.25 (1.24, 1.26)** | **1.25 (1.24, 1.27)** | |
| 30-34 | 232.2 | 56.4 | **1.32 (1.31, 1.33)** | **1.27 (1.26, 1.28)** | **1.28 (1.27, 1.28)** | |
| ≥35 | 230.0 | 57.2 | **1.33 (1.32, 1.34)** | **1.26 (1.25, 1.27)** | **1.27 (1.26, 1.28)** | |
| Region of residence, n (%) |  |  |  |  |  | |
| Urban | 237.0 | 60.5 | **1.34 (1.33, 1.35)** | **1.29 (1.28, 1.30)** | **1.29 (1.28, 1.30)** | |
| Rural | 246.6 | 58.1 | **1.31 (1.30, 1.32)** | **1.27 (1.26, 1.28)** | **1.28 (1.27, 1.28)** | |
| Income level, n (%) |  |  |  |  |  | |
| 1st quartile | 279.1 | 71.2 | **1.34 (1.33, 1.35)** | **1.30 (1.29, 1.31)** | **1.30 (1.29, 1.31)** | |
| 2nd quartile | 251.8 | 59.4 | **1.31 (1.30, 1.32)** | **1.25 (1.24, 1.26)** | **1.26 (1.25, 1.27)** | |
| 3rd quartile | 231.2 | 55.6 | **1.32 (1.31, 1.33)** | **1.26 (1.25, 1.27)** | **1.27 (1.26, 1.28)** | |
| 4th quartile | 201.3 | 48.6 | **1.32 (1.31, 1.33)** | **1.27 (1.25, 1.28)** | **1.28 (1.26, 1.29)** | |
| Parity, n (%) |  |  |  |  |  | |
| 1 | 239.5 | 58.6 | **1.32 (1.32, 1.33)** | **1.29 (1.28, 1.29)** | **1.29 (1.28, 1.30)** | |
| ≥2 | 245.1 | 59.7 | **1.32 (1.31, 1.33)** | **1.26 (1.25, 1.27)** | **1.26 (1.26, 1.27)** | |
| SMM, n (%) |  |  |  |  |  | |
| 0 | 235.4 | 56.6 | **1.32 (1.31, 1.32)** | **1.28 (1.28, 1.29)** | **1.29 (1.28, 1.29)** | |
| 1 | 302.9 | 76.9 | **1.34 (1.32, 1.36)** | **1.27 (1.25, 1.28)** | **1.27 (1.25, 1.28)** | |
| ≥2 | 362.3 | 102.2 | **1.39 (1.32, 1.47)** | **1.36 (1.29, 1.44)** | **1.37 (1.30, 1.44)** | |
| Delivery type, n (%) |  |  |  |  |  | |
| Vaginal delivery | 222.9 | 53.4 | **1.32 (1.31, 1.32)** | **1.26 (1.25, 1.26)** | **1.26 (1.25, 1.27)** | |
| Cesarean section | 265.3 | 65.6 | **1.33 (1.32, 1.34)** | **1.28 (1.28, 1.29)** | **1.29 (1.28, 1.30)** | |
| Number of hospital admissions in a year before pregnancy, n (%) |  |  |  |  |  | |
| 0 | 221.2 | 48.8 | **1.28 (1.28, 1.29)** | **1.23 (1.23, 1.24)** | **1.24 (1.24, 1.25)** | |
| 1 | 284.9 | 76.2 | **1.37 (1.35, 1.38)** | **1.29 (1.28, 1.30)** | **1.29 (1.28, 1.31)** | |
| ≥2 | 443.1 | 155.7 | **1.54 (1.52, 1.56)** | **1.49 (1.47, 1.51)** | **1.51 (1.48, 1.53)** | |
| Number of outpatient contacts in a year before pregnancy, n (%) |  |  |  |  |  | |
| 0 | 167.9 | 41.9 | **1.33 (1.29, 1.38)** | **1.30 (1.26, 1.35)** | **1.29 (1.25, 1.34)** | |
| 1 | 162.2 | 33.2 | **1.26 (1.22, 1.30)** | **1.24 (1.20, 1.28)** | **1.23 (1.19, 1.28)** | |
| ≥2 | 246.4 | 60.3 | **1.32 (1.32, 1.33)** | **1.28 (1.28, 1.29)** | **1.29 (1.28, 1.29)** | |
| History of neuropsychiatric disorder, n (%) |  |  |  |  |  | |
| No | 228.0 | 54.2 | **1.31 (1.31, 1.32)** | **1.27 (1.26, 1.27)** | **1.27 (1.27, 1.28)** | |
| Yes | 349.1 | 90.9 | **1.35 (1.34, 1.37)** | **1.29 (1.28, 1.31)** | **1.30 (1.28, 1.31)** | |
| History of substance use disorder, n (%) |  |  |  |  |  | |
| No | 242.2 | 59.2 | **1.32 (1.32, 1.33)** | **1.28 (1.28, 1.29)** | **1.29 (1.28, 1.29)** | |
| Yes | 346.8 | 17.8 | 1.05 (0.98, 1.13) | 0.97 (0.90, 1.05) | 0.97 (0.90, 1.05) | |
| Plurality, n (%) |  |  |  |  |  | |
| Singleton | 242.2 | 59.1 | **1.32 (1.32, 1.33)** | **1.29 (1.28, 1.29)** | **1.29 (1.28, 1.29)** | |
| Multiple birth | 248.9 | 49.8 | **1.25 (1.22, 1.28)** | **1.30 (1.26, 1.33)** | **1.29 (1.26, 1.33)** | |
| **Infant characteristics** |  |  |  |  |  | |
| Infant sex, n (%) |  |  |  |  |  | |
| Male | 255.7 | 59.5 | **1.30 (1.30, 1.31)** | **1.27 (1.26, 1.28)** | **1.27 (1.27, 1.28)** | |
| Female | 228.4 | 58.7 | **1.35 (1.34, 1.35)** | **1.29 (1.28, 1.29)** | **1.29 (1.29, 1.30)** | |
| Birth season, n (%) |  |  |  |  |  | |
| Spring | 243.7 | 64.1 | **1.36 (1.34, 1.37)** | **1.30 (1.29, 1.31)** | **1.31 (1.29, 1.32)** | |
| Summer | 240.5 | 56.8 | **1.31 (1.30, 1.32)** | **1.25 (1.24, 1.26)** | **1.26 (1.25, 1.27)** | |
| Autumn | 254.5 | 64.5 | **1.34 (1.33, 1.35)** | **1.30 (1.29, 1.32)** | **1.31 (1.30, 1.32)** | |
| Winter | 231.6 | 51.8 | **1.29 (1.28, 1.30)** | **1.24 (1.23, 1.25)** | **1.24 (1.23, 1.26)** | |
| Year of delivery, n (%) |  |  |  |  |  | |
| 2010 to 2012 | 192.5 | 46.6 | **1.32 (1.31, 1.33)** | **1.26 (1.26, 1.27)** | **1.27 (1.26, 1.28)** | |
| 2013 to 2015 | 255.4 | 66.1 | **1.35 (1.34, 1.36)** | **1.28 (1.27, 1.29)** | **1.29 (1.28, 1.30)** | |
| 2016 to 2017 | 317.5 | 73.8 | **1.30 (1.29, 1.31)** | **1.26 (1.25, 1.27)** | **1.27 (1.26, 1.28)** | |
| At-risk newborn, n (%) |  |  |  |  |  | |
| Preterm birth | 347.9 | 92.1 | **1.36 (1.34, 1.38)** | **1.34 (1.32, 1.36)** | **1.35 (1.33, 1.38)** | |
| Low birth weight | 342.7 | 89.9 | **1.36 (1.33, 1.38)** | **1.35 (1.32, 1.38)** | **1.37 (1.34, 1.39)** | |

*Abbreviation:* IRR, incidence rate ratio; CI, confidence interval; PS, propensity score; SMM, severe maternal morbidity.

Numbers in bold indicate significant differences (*p*<0.05).

^*^ Incidence rates were calculated as the number of hospital admissions per 1,000 person-years. Rate differences represent the absolute difference in incidence rates between the opioid exposed and unexposed groups per 1,000 person-years.

^†^ Crude estimates were unadjusted.

**^a^ Model 1 was adjusted for maternal age at delivery; infant sex; region of residence (urban and rural); household income level (1st, 2nd, 3rd, and 4th quartiles); birth season (spring, summer, autumn, and winter); parity (1 and ≥2); SMM score;** number of hospital outpatient visits and admissions in the year before pregnancy**; preterm birth; low birth weight; delivery type (vaginal delivery or cesarean section); and year of delivery (2010-2012, 2013-2015, and 2016-2017).**

**^b^ Model 2 was adjusted for all variables in Model 1,** with further adjustment for maternal history of substance use disorders and neuropsychiatric disorders, and plurality (number of infants per delivery).
